# Performance of the American Heart Association’s PREVENT Equations Among Disaggregated Racial and Ethnic Subgroups

**DOI:** 10.1001/jamacardio.2025.1865

**Published:** 2025-06-25

**Authors:** Xiaowei Yan, Adrian M. Bacong, Qiwen Huang, Hannah Husby, Ramzi Dudum, Powell Jose, Latha Palaniappan, Fatima Rodriguez

**Affiliations:** 1 Center for Health Systems Research, Sutter Health, Walnut Creek, California; 2 Palo Alto Medical Foundation Institute, Sutter Health, Palo Alto, California; 3 Division of Cardiovascular Medicine and the Cardiovascular Institute, School of Medicine, Stanford University, Stanford, California; 4 Division of Cardiology, University of California, San Francisco; 5 Sutter Medical Group, Sutter Health, Sacramento, California; 6 Stanford Center for Asian Health Research and Education (CARE), Stanford University, Sacramento, California; 7 Center for Digital Health, Stanford University School of Medicine, Sacramento, California

## Abstract

**Importance:**

In the original validation, the Predicting Risk of Cardiovascular Disease (CVD) Events (PREVENT) equations demonstrated good discrimination and calibration among racial and ethnic groups, but the model performance among Asian and Hispanic disaggregated subgroups has not been previously described.

**Objective:**

To assess the performance of the PREVENT equations by race and ethnicity, including disaggregated Asian and Hispanic subgroups.

**Design, Setting, and Participants:**

This was an electronic health record–based retrospective cohort study of primary care patients aged 30 to 79 years across Sutter Health, a large integrated health system in Northern California, from January 2010 to September 2023. Patients who had at least 2 primary care visits during the study period were eligible for the study (1 484 582). Those outside of the study age range, with prior CVD events in the washout period, missing key predictors, or having at least 1 predictor out of the allowed normal range for the American Heart Association’s PREVENT equations, were excluded, leaving a study population of 361 778.

**Exposure:**

Eligible patients had complete baseline data required for the PREVENT equations, including non–high-density lipoprotein cholesterol, high-density lipoprotein cholesterol, systolic blood pressure, body mass index, estimated glomerular filtration rate (or creatinine), diabetes, and current smoking status, and were free from CVD at baseline.

**Main Outcomes and Measures:**

The primary outcomes were CVD events, identified using *International Classification of Diseases*, *Ninth *and* Tenth Revisions*, codes described in the PREVENT derivation.

**Results:**

Among 361 778 patients who met the inclusion criteria, mean (SD) age was 54.6 (12.2) years; 191 151 (53%) were female; and 81 424 (22%) were non-Hispanic Asian and 40 897 (11%) were Hispanic. Over a mean (SD) follow-up of 8.1 (3.2) years, there were 22 648 (6.3%) CVD events. The C statistic for total CVD was 0.83 (95% CI, 0.82-0.84) for the Asian population and 0.80 (95% CI, 0.79-0.81) for the Hispanic population. The calibration slopes were 0.84 (95% CI, 0.78-0.90) and 1.02 (95% CI, 0.94-1.10) for Asian and Hispanic patients, respectively. Within the Asian population, C statistics for total CVD among disaggregated Asian subgroups ranged from 0.79 (95% CI, 0.77-0.81) in Filipino patients to 0.85 (95% CI, 0.83-0.87) in Asian Indian patients. The calibration slope for total CVD was less than 1 for all Asian subgroups except Asian Indian. Among disaggregated Hispanic subgroups, the C statistics were similar and between 0.80 and 0.82 for total CVD, and the calibration slope for total CVD included 1 for all subgroups. There were small differences in the performance of atherosclerotic CVD and heart failure PREVENT equations among racial and ethnic groups and subgroups.

**Conclusions and Relevance:**

The PREVENT equations appropriately predicted risk in contemporary diverse Asian and Hispanic subgroups with modest variation in performance across disaggregated subgroups.

## Key Points

**Question:**

How do the Predicting Risk of Cardiovascular disease (CVD) Events (PREVENT) equations perform in disaggregated Asian and Hispanic groups?

**Findings:**

In this cohort study of 361 778 primary care patients (33% Asian or Hispanic), the risk of incident CVD differed significantly by race and ethnicity. The PREVENT CVD, atherosclerotic CVD (ASCVD), and heart failure equations performed well, and the PREVENT ASCVD equations outperformed the pooled cohort equations overall and among racial and ethnic groups and subgroups.

**Meaning:**

The PREVENT equations accurately predicted CVD, ASCVD, and heart failure events across diverse Asian and Hispanic patient populations.

## Teaser

This cohort study examines the performance of the American Heart Association’s Predicting Risk of Cardiovascular Disease Events equations among racial and ethnic subgroups.

## Introduction

Risk assessment is foundational to identifying at-risk primary prevention individuals who might benefit from preventive therapies.^1^ The American Heart Association (AHA)’s Predicting Risk of Cardiovascular Disease (CVD) Events (PREVENT) equations were introduced as a new method for cardiovascular risk assessment and include separate equations to predict 10-year and 30-year risk of CVD events (ie, total CVD, atherosclerotic CVD [ASCVD], and heart failure [HF]).^2,3^ PREVENT addressed limitations of previous pooled cohort equations (PCE),^3,4^ including concerns that PCE overestimated ASCVD risk, desires to replace a race-specific model with a race-free model,^4^ and the incorporation of kidney-related risk factors, such as estimated glomerular filtration rate and urinary albumin to creatinine ratio, to recognize the interplay of cardiovascular-kidney-metabolic conditions.^3^ These updates intend to provide a comprehensive, more generalizable, and less biased risk assessment tool to guide clinical management decisions in primary prevention of CVD.

While non-Hispanic Asian and Hispanic individuals comprise 6% and 19% of the US population, respectively,^5^ Asian (83 602 [3%]) and Hispanic (186 894 [5%]) groups were underrepresented in the PREVENT equation development study.^2^ These populations are projected to continue growing and will likely be among the largest racial and ethnic groups in 2060.^6^ However, both comprise heterogenous subgroups with different cardiometabolic risk factors and outcomes.^7^ Therefore, the primary objective of this study was to examine the performance of the PREVENT equations in a large, multiethnic health care system where Asian and Hispanic groups are overrepresented compared to the model development and validation cohort, and further assess the equations across disaggregated Asian and Hispanic groups using longitudinal electronic health record (EHR) data.

## Methods

### Study Population

We examined the primary care population at Sutter Health—a large, diverse health system in Northern California. Patients who had 2 or more primary care visits on different dates from January 1, 2010, to September 30, 2023, were eligible. A 2-year period after the first primary care visit was taken as a washout period, and the index date was defined as 2 years after the first primary care visit date. Patients were included if they were aged 30 to 79 years and were free of CVD within the washout period.^2^ Eligible patients were also required to have at least 1 primary care visit after the index date, complete baseline data recorded within the 2-year washout period, and baseline data within the ranges required for the PREVENT equations, including total cholesterol ranging from 130 to 320 mg/dL (used to calculate non–high-density lipoprotein cholesterol [non–HDL-C]), HDL-C ranging from 20 to 100 mg/dL, systolic blood pressure (SBP) ranging from 90 to 200 mm Hg, and body mass index (BMI; calculated as weight in kilograms divided by height in meters squared) ranging from 18.5 to less than 40.0.

### Data Collection at Baseline

Predictors included in the PREVENT equations (age, sex, non–HDL-C, HDL-C, SBP, diabetes status, current smoking status, antihypertensive medication, statin medication, and estimated glomerular filtration rate) were collected at baseline. If multiple measures were available for a predictor, the measurement closest to the index date was used.

Self-reported race and ethnicity were first grouped into non-Hispanic Asian (hereafter, Asian), non-Hispanic Black (hereafter, Black), Hispanic, non-Hispanic White (hereafter, White), and non-Hispanic other (hereafter, other), including American Indian or Alaska Native, Native Hawaii or Other Pacific Islander, multiple race categories, or those who selected other. Missing or unknown race and ethnicity information (ie, not reported or marked as “prefer not to answer” or “unknown”) was coded as an unknown category. If a patient reported non-Hispanic and multiple race categories, we assigned patients to a multiple race group, and consolidated them into the other group because of small numbers. Asian and Hispanic patients were further disaggregated by self-reported region of origin. Asian groups included Asian Indian, Chinese, Japanese, Korean, Filipino, Vietnamese, Indonesian, Laotian, Nepalese, and others. Hispanic groups included Mexican, Costa Rican, Cuban, and others. We consolidated groups that were too small for analysis (less than 200 individuals) based on patients’ responses into “other Asian” or “other Hispanic.” Detailed information on the participants in these groups can be found in the eMethods in Supplement 1.

### Outcome Definition

CVD events were identified using *International Classification of Diseases*, *Ninth *and* Tenth Revisions* (*ICD-9* and *ICD-10*, heretoforward *ICD*), codes described in the PREVENT derivation.^2,3^ Patients with any one of these *ICD* diagnosis codes in their outpatient, emergency department, or hospitalization diagnoses were defined as having a CVD event (eMethods in Supplement 1). CareEverywhere data were incorporated into EHR data to capture encounters and diagnosis occurring outside of the study health care system. A 2-year washout period was used to define incident CVD cases, as this duration has been shown to reduce misclassification due to delayed diagnosis, ensure exclusion of subclinical disease, and align with prior studies on cardiovascular risk estimation.^8,9,10^ An incident CVD event was defined as the first diagnosed CVD in the study period without any CVD events in the 2-year washout period. If multiple CVD events occurred during the follow-up for an event of interest, the follow-up time ended at either the earliest occurrence of the event or the earliest date among all-cause death, end of study, or 10-year postindex date.

Cause of death (ie, CVD death) was identified for participants for whom the reason for death was documented in the EHR or if patients died during hospitalization. State death records have been integrated into the EHR since 2015, and associated *ICD* codes were created based on reason for death if available. The *ICD* codes that recorded reason for death were used to identify a CVD-related death. A CVD-related death was taken as a CVD event. The *ICD* codes and codes that were used to extract predictors are documented in the eMethods in Supplement 1.

### Statistical Analysis

Summary statistics of patient characteristics were created for each race and ethnic group separately, followed by disaggregated Asian and Hispanic subgroups. The ratio of predicted events from PREVENT equations with observed events for each outcome type (eg, total CVD, ASCVD, and HF) was estimated as a relative ratio and statistically compared using a paired *t* test. Paired *t* tests were conducted for each race and ethnic group and disaggregated Asian and Hispanic subgroups.

Model performance was assessed with the Harrel C statistic, and confidence intervals were estimated based on the bootstrapping method with 500 bootstraps. For calibration measures, the Homser-Lemeshow test was used to partition estimated risk into data-driven deciles, and a calibration slope was estimated based on linear regression across deciles. If there were fewer than 10 samples in a decile, then the samples from that decile were combined with the next decile. A calibration slope of 1.0 indicates optimal calibration, while a slope of less than 1.0 indicates lower observed than predicted risk (ie, overprediction), and a slope greater than 1.0 indicates higher observed than predicted risk (ie, underprediction). A calibration curve was produced for the overall cohort, for each race and ethnic group, and for each disaggregated Asian and Hispanic subgroup. These analyses were repeated for all end points (total CVD, ASCVD, and HF events).

For patients aged 40 to 79 years who are eligible for PREVENT ASCVD equation and PCE for an ASCVD event, we estimated the Harrel C statistics (and 95% CI) and calibration slope (and 95% CI) based on PCE model and PREVENT ASCVD equation, respectively.

## Results

### Study Population

**Figure 1.**
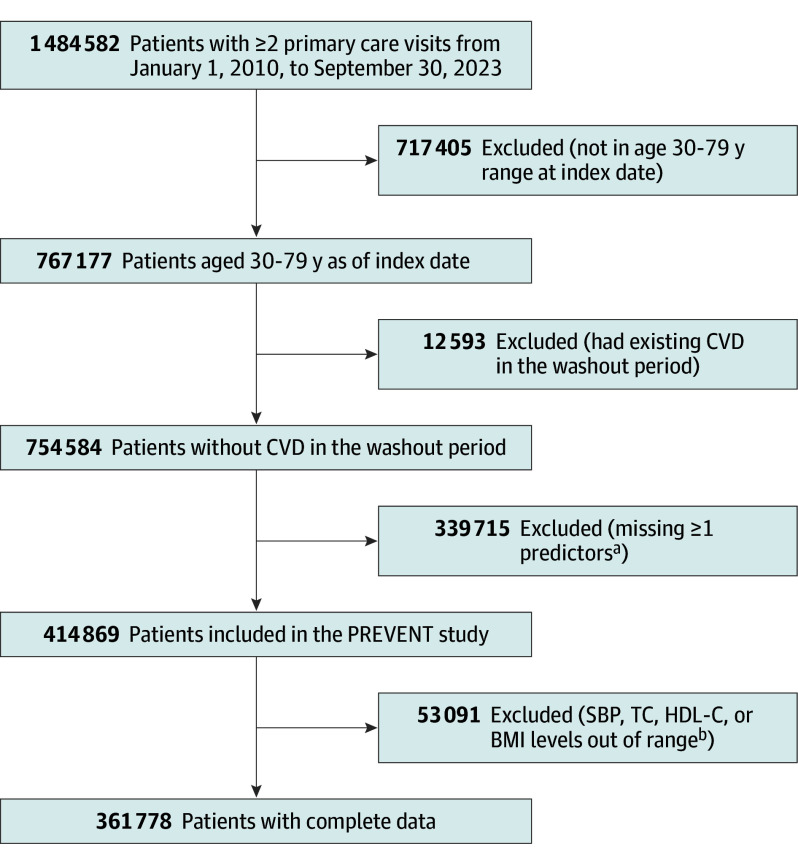
Study Cohort Flow Diagram BMI indicates body mass index; CVD, cardiovascular disease; HDL-C, high-density lipoprotein cholesterol; PREVENT, Predicting Risk of CVD Events; SBP, systolic blood pressure; TC, total cholesterol. ^a^Missing TC (n = 269 910); missing HDL-C (n = 223 230), missing SBP (n = 415 201), missing BMI (n = 22 750), missing smoking status (n = 43 160), missing creatinine (n = 237 865), missing sex (n = 43 160). There were 99 757 participants missing 1 predictor, 49 851 missing 2 predictors, and 190 107 missing more than 3 predictors. ^b^TC range: 130-320 mg/dL; HDL-C range: 20-100 mg/dL; SBP range, 90-200 mm Hg; BMI (calculated as weight in kilograms divided by height in meters squared) range, 18.5-40.0.

Figure 1 displays the flowchart of the derived study sample. More than 1.4 million primary care patients were identified within the study period; 717 405 were excluded due to age (ie, outside of the 30-79–year range) and 12 593 patients were excluded due to prior CVD events in the washout period. Among 754 584 remaining patients, 339 715 patients were further excluded due to missing one or more key predictors, and 53 091 were excluded due to at least 1 predictor out of the allowed normal range for the AHA PREVENT equations. The final study included 361 778 patients.

Among these, the mean (SD) age was 53.1 (12.6) years; 191 151 (52.8%) were female (Table 1). More than 22% of patients (81 424) were Asian, and 11.3% (40 897) were Hispanic. Compared with other racial or ethnic groups, Asian were younger (mean [SD] age, 47.9 [12.2] years) (Table 2), had the lowest use of antihypertensive medications (26.9% vs more than 36%) and statins (18.3% vs more than 25%), and had lower BMI (mean [SD], 25.7 [4.0] vs more than 27), smoking (3.8% vs more than 5%), and SBP (mean [SD], 118.8 [15.5] vs more than 121 mm Hg) (Table 2).

**Table 1.** Baseline Characteristics for Sutter Patient Cohort and the Predicting Risk of Cardiovascular Disease (CVD) Events (PREVENT) Original Validation Cohort

Characteristic	No. (%)			
	Sutter cohort (N = 361 778)		PREVENT cohort (N = 3 330 085)^a^	
	Female	Male	Female	Male
Total, No.	191 151	170 627	1 894 882	1 435 203
Age, mean (SD), y	53.6 (12.8)	52.3 (12.4)	52 (13.0)	52 (12.0)
Race and ethnicity				
Non-Hispanic Asian	43 009 (22.5)	38 414 (22.5)	51 162 (2.7)	31 574 (2.2)
Non-Hispanic Black	6881 (3.6)	5293 (3.1)	189 488 (10.0)	117 687 (8.2)
Hispanic	22 747 (11.9)	18 097 (10.6)	79 585 (4.2)	53 103 (3.7)
Non-Hispanic White	101 883 (53.3)	91 510 (53.6)	1 478 008 (78.0)	1 148 162 (80.0)
Other or missing	16 630 (8.7)	17 243 (10.1)	92 849 (4.9)	78 936 (5.5)
Cardiovascular risk factors/predictors				
SBP, mean (SD), mm Hg	121 (16)	125 (15)	123 (16)	128 (15)
TC, mean (SD), mmol/L	5 (0.9)	4.9 (0.9)	5.0 (0.8)	4.9 (0.8)
Non–HDL-C, mean (SD), mmol/L^b^	3.5 (0.9)	3.6 (0.9)	3.4 (0.8)	3.6 (0.8)
HDL-C, mean (SD), mmol/L	1.6 (0.4)	1.3 (0.3)	1.5 (0.4)	1.2 (0.3)
BMI, mean (SD)	27.3 (5.0)	28.3 (4.2)	28 (5.0)	29 (4.0)
Diabetes	18 159 (9.5)	19 110 (11.2)	208 437 (11.0)	186 576 (13.0)
Current smoking	9749 (5.1)	14 162 (8.3)	89 059 (4.7)	70 325 (4.9)
Antihypertensive treatment	66 138 (34.6)	61 938 (36.3)	454 772 (24.0)	416 209 (29.0)
Statin treatment	42 244 (22.1)	47 434 (27.8)	265 283 (14.0)	243 985 (17.0)
Estimated glomerular filtration rate, mean (SD), mL/min/1.73 m^2^	90 (20)	90 (18)	91 (18)	91 (17)
Add-on risk factors/predictors in optional models				
Baseline UACR	17 204 (9.0)	18 598 (10.9)	NA	NA
UACR, median (IQR), mg/g	11 (7-23)	10 (5-26)	8 (8-12)	8 (8-11)
HbA_1c_ among those with diabetes, mean (SD)	7.17 (1.46)	7.25 (1.51)	7.2 (1.80)	7.6 (1.90)
HbA_1c_ among those without diabetes, mean (SD)	5.69 (0.71)	5.75 (0.65)	5.50 (0.60)	5.60 (0.80)
Follow-up time, mean (SD), y	8.3 (3.2)	7.9 (3.2)	5.0 (3.2)	4.9 (3.2)
Outcome in follow-up	Outcomes within 10-y follow-up period		During the whole follow-up period	
Total CVD	10 704 (5.6)	11 944 (7.0)	54 952 (2.9)	50 232 (3.5)
ASCVD	6117 (3.2)	7166 (4.2)	34 108 (1.8)	34 445 (2.4)
Heart failure	6117 (3.2)	6484 (3.8)	30 318 (1.6)	25 834 (1.8)
Death in 10-y follow-up	3632 (1.9)	3754 (2.2)	81 480 (4.3)	76 066 (5.3)

Abbreviations: ASCVD, atherosclerotic cardiovascular disease; BMI, body mass index (calculated as weight in kilograms divided by height in meters squared); HbA_1c_, hemoglobin A_1c_; HDL-C, high-density lipoprotein cholesterol; NA, not applicable; SBP, systolic blood pressure; TC, total cholesterol; UACR, urinary albumin to creatinine ratio.

^a^ From Khan et al.^2^

^b^ Non–HDL-C = TC − HDL-C.

**Table 2.** Baseline Demographic and Clinical Characteristics, Stratified by Race and Ethnicity Groups in the Sutter Cohort

Category	No. (%)					
	Non-Hispanic Asian	Non-Hispanic Black	Hispanic	Non-Hispanic White	Non-Hispanic other	Unknown
No. (% of group)	81 424 (22.5)	12 134 (3.4)	40 897 (11.3)	193 337 (53.4)	11 600 (3.2)	22 386 (6.2)
Age, mean (SD), y	47.9 (12.2)	53.2 (11.9)	50.6 (12.3)	56.2 (12.0)	51.7 (12.6)	48.9 (12.1)
Sex						
Female	42 992 (52.8)	6880 (56.7)	22 780 (55.7)	110 889 (52.7)	6171 (53.2)	10 499 (46.9)
Male	38 432 (47.2)	5254 (43.3)	18 117 (44.3)	91 448 (47.3)	5429 (46.8)	11 887 (53.1)
BMI, mean (SD)	25.7 (4.0)	30 (4.7)	29.5 (4.6)	28.1 (4.7)	28.5 (4.8)	27.2 (4.5)
Current smoking	3094 (3.8)	1371 (1.3)	2699 (6.6)	14 500 (7.5)	1067 (9.2)	1298 (5.8)
Lipid panel, mean (SD)						
TC, mmol/L	4.9 (0.8)	4.9 (0.9)	4.9 (0.9)	4.9 (0.9)	4.9 (0.9)	4.9 (0.9)
HDL-C, mmol/L	1.4 (0.4)	1.5 (0.4)	1.3 (0.4)	1.4 (0.4)	1.4 (0.4)	1.4 (0.4)
Creatinine, mg/dL	0.83 (0.22)	0.99 (0.27)	0.84 (0.23)	0.92 (0.22)	0.88 (0.24)	0.86 (0.22)
eGFR from creatinine	98.3 (17.3)	81.3 (19.2)	95 (18.1)	85.6 (17.7)	91.7 (18.8)	95.8 (17.8)
SBP, mean (SD)	118.8 (15.5)	127.4 (16.0)	123.2 (15.5)	124.4 (15.6)	123.2 (15.9)	121 (15.8)
T2DB	8875 (10.9)	2087 (17.2)	6462 (15.8)	16 240 (8.4)	1636 (14.1)	1925 (8.6)
Antihypertensive medication order	21 903 (26.9)	6261 (51.6)	14 273 (34.9)	75 595 (39.1)	4234 (36.5)	5820 (26.0)
Statin medication order	14 901 (18.3)	3118 (25.7)	10 102 (24.7)	54 521 (28.2)	3028 (26.1)	4052 (18.1)
Primary insurance, No. (%						
HMO	15 796 (19.4)	2427 (20.0)	8466 (20.7)	30 741 (15.9)	2088 (18.0)	4186 (18.7)
Medicaid/Medi-Cal	1059 (1.3)	303 (2.5)	941 (2.3)	1933 (1.0)	302 (2.6)	224 (1.0)
Medicare FFS	6840 (8.4)	1869 (15.4)	4662 (11.4)	36 541 (18.9)	1601 (13.8)	2082 (9.3)
Medicare HMO	1384 (1.7)	522 (4.3)	1554 (3.8)	9860 (5.1)	441 (3.8)	381 (1.7)
PPO/FFS	52 193 (64.1)	5315 (43.8)	20 408 (49.9)	87 582 (45.3)	5707 (49.2)	13 946 (62.3)
Other government/self/other/unknown	4071 (5.0)	1699 (14.0)	4867 (11.9)	26 487 (13.7)	1462 (12.6)	1567 (7.0)
UACR test in baseline	7572 (9.3)	2233 (18.4)	6135 (15.0)	16 434 (8.5)	1601 (13.8)	1858 (8.3)
UACR, median (IQR), mg/g	12 (7-29)	10 (6-27)	11 (6-28)	9 (6-21)	11 (6-28)	11 (7-26)
Follow-up time, mean (SD), y	8 (3.2)	7.8 (3.3)	7.7 (3.3)	8.4 (3.1)	7.5 (3.3)	6.6 (3.5)
Outcome in follow-up						
Total CVD	2606 (3.2)	1092 (9.0)	2249 (5.5)	15 274 (7.9)	754 (6.5)	604 (2.7)
ASCVD	1710 (2.1)	631 (5.2)	1431 (3.5)	8700 (4.5)	441 (3.8)	381 (1.7)
Heart failure	1140 (1.4)	680 (5.6)	1145 (2.8)	8894 (4.6)	452 (3.9)	291 (1.3)
Death in 10-y follow-up, No./total No. (%)	733 (0.9)	352 (2.9)	695 (1.7)	5220 (2.7)	244 (2.1)	246 (1.1)
PREVENT model performance						
C statistic (95% CI)						
Total CVD	0.83 (0.82-0.84)	0.77 (0.76-0.79)	0.80 (0.80-0.81)	0.79 (0.78-0.80)	0.82 (0.81-0.84)	0.84 (0.83-0.86)
ASCVD	0.80 (0.79-0.81)	0.76 (0.75-0.78)	0.78 (0.77-0.79)	0.76 (0.75-0.76)	0.79 (0.77-0.81)	0.81 (0.79-0.83)
Heart failure	0.88 (0.87-0.89)	0.79 (0.77-0.80)	0.84 (0.83-0.86)	0.82 (0.81-0.82)	0.85 (0.84-0.87)	0.89 (0.87-0.90)
Calibration curve slope (95% CI)						
Total CVD	0.84 (0.78-0.90)	1.16 (1.09-1.23)	1.02 (0.94-1.10)	1.18 (1.11-1.25)	1.14 (1.04-1.23)	0.71 (0.66-0.75)
ASCVD	0.86 (0.80-0.92)	1.21 (1.10-1.33)	1.03 (0.96-1.10)	1.08 (1.04-1.12)	1.03 (0.99-1.07)	0.66 (0.61-0.71)
Heart failure	0.79 (0.69-0.89)	1.10 (1.04-1.16)	0.98 (0.88-1.07)	1.19 (1.10-1.27)	1.18 (1.09-1.27)	0.67 (0.61-0.73)

Abbreviations: ASCVD, atherosclerotic cardiovascular disease; BMI, body mass index (calculated as weight in kilograms divided by height in meters squared); CVD, cardiovascular disease; eGFR, estimated glomerular filtration rate; FFS, fee-for-service; HDL-C, high-density lipoprotein cholesterol; HMO, health maintenance organization; PPO, preferred provider organization; PREVENT, Predicting Risk of CVD Events; SBP, systolic blood pressure; T2DB, type 2 diabetes; TC, total cholesterol; UACR, urinary albumin to creatinine ratio.

Among 81 424 Asian patients (Table 3), the major subgroups included Asian Indian (25 182 [31%]), Chinese (24 313 [30%]), Filipino (11 539 [14%]), Japanese (4174 [5%]), Korean (2369 [3%]), and Vietnamese (2369 [3%]), and other Asian (11 478 [14%]). Asian Indian participants were youngest (mean [SD] age, 42.8 [10.1] years) while Japanese were oldest (mean [SD] age, 55.8 [11.9] years).

**Table 3.** Baseline Patient Characteristics by Disaggregated Hispanic and Asian Subgroups in the Sutter Cohort

Characteristic	No. (%)									
	Hispanic			Non-Hispanic Asian						
	Mexican (n = 19 528)	Puerto Rican (n = 861)	Other Hispanic (n = 20 508)	Asian Indian (n = 25 182)	Chinese (n = 24 313)	Filipino (n = 11 539)	Japanese (n = 4174)	Korean (n = 2369)	Vietnamese (n = 2369)	Other Asian (n = 11 478)
Age, mean (SD), y	50 (12.4)	51.7 (11.8)	51 (12.3)	42.8 (10.1)	50.3 (12.3)	50.6 (12.1)	55.8 (11.9)	48 (11.9)	48.2 (11.3)	48.2 (12.3)
Sex										
Female	10 662 (54.6)	477 (55.4)	11 649 (56.8)	11 130 (44.2)	13 032 (53.6)	7143 (61.9)	2521 (60.4)	1324 (55.9)	1395 (58.9)	6451 (56.2)
Male	8866 (45.4)	384 (44.6)	8859 (43.2)	14 052 (55.8)	11 281 (46.4)	4396 (38.1)	1653 (39.6)	1045 (44.1)	974 (41.1)	5027 (43.8)
BMI, mean (SD)	29.9 (4.5)	29.3 (4.7)	29.2 (4.7)	26.3 (3.8)	24.5 (3.5)	27.2 (4.2)	26 (4.3)	25 (3.8)	24.3 (3.4)	25.7 (4.1)
Current smoking	1250 (6.4)	80 (9.3)	1354 (6.6)	856 (3.4)	535 (2.2)	750 (6.5)	196 (4.7)	142 (6.0)	97 (4.1)	517 (4.5)
Lipid panel, mean (SD)										
TC, mmol/L	4.90 (0.87)	4.92 (0.85)	4.93 (0.88)	4.81 (0.81)	4.90 (0.82)	4.90 (0.87)	5.03 (0.85)	4.96 (0.83)	4.98 (0.82)	4.92 (0.85)
HDL-C, mmol/L	1.30 (0.36)	1.38 (0.40)	1.36 (0.38)	1.22 (0.33)	1.46 (0.38)	1.42 (0.38)	1.55 (0.41)	1.47 (0.40)	1.46 (0.38)	1.44 (0.39)
Creatinine, mg/dL	0.84 (0.22)	0.90 (0.23)	0.84 (0.23)	0.84 (0.21)	0.82 (0.22)	0.85 (0.25)	0.84 (0.22)	0.79 (0.22)	0.8 (0.23)	0.82 (0.23)
eGFR from creatinine	95.8 (18.0)	89.2 (18.1)	94.5 (18)	102 (16.4)	97.3 (16.6)	93.8 (18.9)	91 (17.3)	101.4 (16.0)	99.8 (16.4)	98.5 (17.4)
SBP, mean (SD)	123.4 (15.4)	123.8 (16.2)	123 (15.5)	117 (14.7)	117.4 (15.4)	124.2 (15.7)	122.7 (16.0)	118.6 (15.1)	117.3 (15.3)	119.4 (15.8)
T2DB	3476 (17.8)	119 (13.8)	2851 (13.9)	2644 (10.5)	1799 (7.4)	2227 (19.3)	576 (13.8)	237 (10.0)	175 (7.4)	1182 (10.3)
Antihypertensive medication order, %	6796 (34.8)	339 (39.4)	7157 (34.9)	4633 (18.4)	5981 (24.6)	5296 (45.9)	1749 (41.9)	599 (25.3)	545 (23.0)	2984 (26.0)
Statin medication order, %	5019 (25.7)	226 (26.2)	4860 (23.7)	3651 (14.5)	3793 (15.6)	3381 (29.3)	1215 (29.1)	400 (16.9)	412 (17.4)	2089 (18.2)
Primary insurance, %										
HMO	4062 (20.8)	155 (18.0)	4266 (20.8)	4356 (17.3)	4814 (19.8)	2573 (22.3)	776 (18.6)	393 (16.6)	573 (24.2)	2319 (20.2)
Medicaid/Medi-Cal	430 (2.2)	15 (1.7)	492 (2.4)	252 (1.0)	292 (1.2)	185 (1.6)	25 (0.6)	33 (1.4)	54 (2.3)	218 (1.9)
Medicare FFS	2109 (10.8)	140 (16.3)	2420 (11.8)	1007 (4.0)	2626 (10.8)	1108 (9.6)	668 (16.0)	246 (10.4)	182 (7.7)	1022 (8.9)
Medicare HMO	683 (3.5)	25 (2.9)	820 (4.0)	151 (0.6)	486 (2.0)	265 (2.3)	179 (4.3)	40 (1.7)	38 (1.6)	253 (2.2)
PPO/FFS	9666 (49.5)	403 (46.8)	10 316 (50.3)	18 836 (74.8)	15 317 (63.0)	6139 (53.2)	2083 (49.9)	1502 (63.4)	1395 (58.9)	6933 (60.4)
Other government/self/other /unknown	2578 (13.2)	123 (14.3)	2194 (10.7)	579 (2.3)	754 (3.1)	1269 (11.0)	442 (10.6)	154 (6.5)	128 (5.4)	746 (6.5)
Baseline UACR	3164 (16.2)	125 (14.5)	2830 (13.8)	1989 (7.9)	1556 (6.4)	1985 (17.2)	476 (11.4)	211 (8.9)	173 (7.3)	1159 (10.1)
UACR, median (IQR), mg/g	11 (6-28)	10 (6-24)	11 (6-29)	10 (6-22)	12 (7-27)	15 (7-44)	13 (7-27)	11 (7-27)	12 (7-30)	13 (7-33)
Follow-up time, mean (SD), y	8 (3.2)	7.9 (3.4)	7.3 (3.4)	8 (3.1)	8.4 (3.0)	8 (3.2)	8.9 (2.9)	7.8 (3.2)	7.9 (3.1)	7 (3.2)
Outcome in follow-up										
Total CVD	1074 (5.5)	44 (5.1)	1128 (5.5)	579 (2.3)	729 (3.0)	531 (4.6)	234 (5.6)	71 (3.0)	69 (2.9)	379 (3.3)
ASCVD	683 (3.5)	25 (2.9)	718 (3.5)	403 (1.6)	486 (2.0)	335 (2.9)	159 (3.8)	54 (2.3)	50 (2.1)	253 (2.2)
Heart failure	547 (2.8)	25 (2.9)	595 (2.9)	302 (1.2)	413 (1.7)	335 (2.9)	129 (3.1)	33 (1.4)	24 (1.0)	264 (2.3)
Death in 10-y follow-up, No./total No. (%)	352 (1.8)	18 (2.1)	328 (1.6)	227 (0.9)	316 (1.3)	277 (2.4)	100 (2.4)	26 (1.1)	26 (1.1)	172 (1.5)
PREVENT model performance										
C statistic (95% CI)										
Total CVD	0.80 (0.79-0.82)	0.82 (0.76-0.88)	0.80 (0.79-0.82)	0.85 (0.83-0.87)	0.83 (0.82-0.84)	0.79 (0.77-0.81)	0.79 (0.76-0.82)	0.82 (0.76-0.87)	0.83 (0.78-0.88)	0.83 (0.81-0.85)
ASCVD	0.78 (0.76-0.80)	0.80 (0.71-0.90)	0.78 (0.77-0.80)	0.82 (0.80-0.84)	0.80 (0.79-0.82)	0.75 (0.72-0.77)	0.76 (0.73-0.80)	0.79 (0.72-0.85)	0.81 (0.75-0.87)	0.81 (0.79-0.84)
Heart failure	0.85 (0.83-0.86)	0.81 (0.75-0.87)	0.84 (0.83-0.86)	0.90 (0.88-0.93)	0.88 (0.86-0.90)	0.85 (0.83-0.88)	0.84 (0.81-0.88)	0.91 (0.86-0.95)	0.87 (0.81-0.94)	0.86 (0.83-0.89)
Calibration curve slope (95% CI)										
Total CVD	1.01 (0.92-1.10)	0.95 (0.54-1.36)	1.04 (0.95-1.13)	0.99 (0.90-1.08)	0.73 (0.67-0.80)	0.77 (0.72-0.82)	0.85 (0.76-0.94)	0.79 (0.64-0.93)	0.86 (0.78-0.95)	0.78 (0.74-0.83)
ASCVD	0.98 (0.91-1.05)	1.07 (0.65-1.49)	1.05 (0.94-1.15)	1.01 (0.94-1.08)	0.77 (0.73-0.82)	0.71 (0.62-0.80)	0.95 (0.86-1.05)	0.91 (0.70-1.12)	0.96 (0.84-1.08)	0.85 (0.76-0.94)
Heart failure	0.95 (0.84-1.07)	0.73 (0.33-1.13)	0.99 (0.92-1.07)	0.92 (0.78-1.07)	0.67 (0.56-0.77)	0.81 (0.74-0.87)	0.68 (0.54-0.82)	0.69 (0.58-0.81)	0.67 (0.60-0.74)	0.70 (0.65-0.76)

Abbreviations: ASCVD, atherosclerotic cardiovascular disease; BMI, body mass index (calculated as weight in kilograms divided by height in meters squared); CVD, cardiovascular disease; eGFR, estimated glomerular filtration rate; FFS, fee-for-service; HDL-C, high density lipoprotein cholesterol; HMO, health maintenance organization; PPO, preferred provider organization; PREVENT, Predicting Risk of CVD Events; SBP, systolic blood pressure; T2DB, type 2 diabetes; TC, total cholesterol; UACR, urinary albumin to creatinine ratio.

Within Hispanic subgroups, 19 528 (48%) were Mexican, followed by Puerto Rican (861 [2%]). Other subgroups were combined into other Hispanic (20 508 [50%]) due to small sample size for each subgroup (Table 3). Compared with subgroups, Puerto Rican patients were more likely to currently smoke, take antihypertensives, statins, and be insured by Medicare fee-for-service.

### Incident CVD Events

During a mean (SD) follow-up of 8.1 (3.2) years, there were 22 648 total CVD events (6.3%) (Table 1). Incident total CVD, ASCVD, and HF events by race and ethnicity groups are shown in Table 2, and for Asian and Hispanic subgroups in Table 3. Compared with White patients (15 274 [7.9%], 8700 [4.5%], and 8894 [4.6%], respectively), Asian (2606 [3.2%], 1710 [2.1%], and 1140 [1.4%]) and Hispanic patients (2249 [5.5%], 1431 [3.5%], and 1145 [2.8%]) had lower incident total CVD, ASCVD, and HF during follow-up. Mortality among Asian and Hispanic groups was 733 (0.9%) and 695 (1.7%), respectively, lower than that for the White group (5220 [2.7%]).

### PREVENT Model Performance

**Figure 2.**
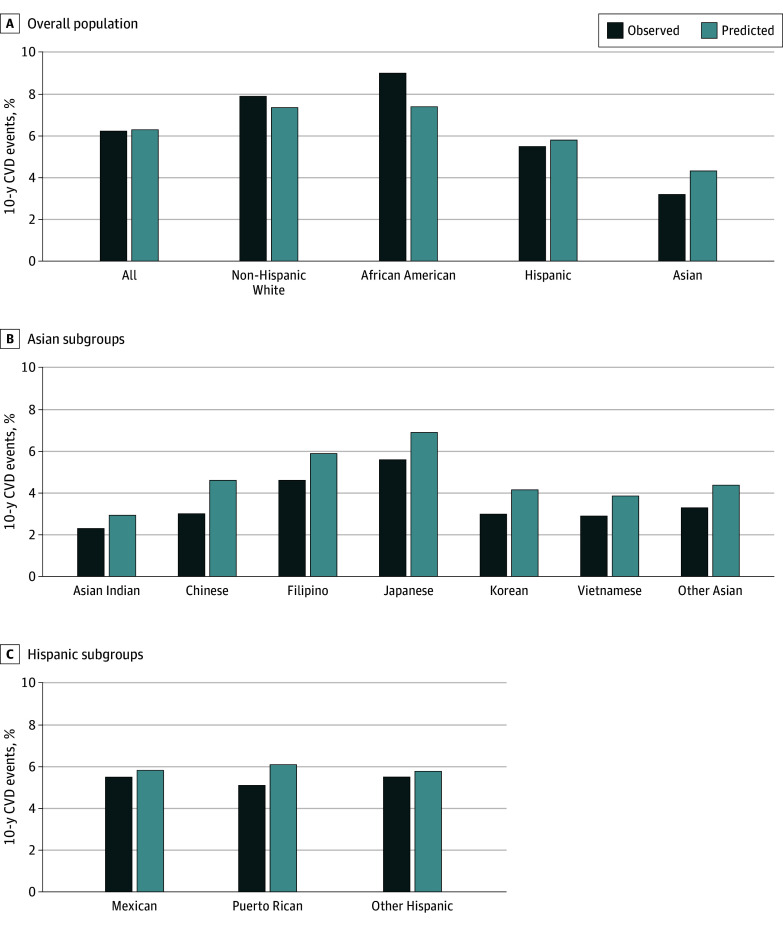
Comparison of 10-Year Observed vs Predicted Risk of Total Cardiovascular Disease (CVD) Outcomes, by Race and Ethnicity and Subgroups

Discrimination and calibration of the PREVENT model by racial and ethnic groups, and disaggregated Asian and Hispanic subgroups are shown in Tables 2 and 3. Predicted vs observed risk for racial and ethnic groups and disaggregated subgroups are shown in Figure 2 (for total CVD) and eFigures 1-2 in Supplement 1 (for ASCVD and HF, respectively). Calibration plots are shown in eFigures 3-11 in Supplement 1.

#### Total CVD

The Harrel C statistics (Table 2) for total CVD were highest for non-the Hispanic Asian group (0.83; 95% CI, 0.82-0.84) and unknown group (0.84; 95% CI, 0.83-0.86), and lowest in the Black group (0.77; 95% CI, 0.76-0.79). The calibration slope varied from 0.84 (95% CI, 0.78-0.90) for the Asian group to 1.18 (95% CI, 1.11-1.25) for the White group, with the best calibration (ie, closest to 1.0) for the Hispanic group (slope, 1.02; 95% CI, 0.94-1.10) (eFigure 3 in Supplement 1), with a predicted risk of 5.8% compared to an observed risk of 5.5% (Figure 2).

Among disaggregated Asian subgroups (Table 3), C statistics ranged from 0.79 (95% CI, 0.77-0.81) among Filipino participants to 0.85 (95% CI, 0.83-0.87) among Asian Indian participants. Among disaggregated Hispanic subgroups, C statistics were similar across all subgroups, ranging from 0.80 to 0.82.

The calibration slopes were generally under 1.0 for all disaggregated Asian subgroups, with the best calibration for Asian Indian participants (slope, 0.99; 95% CI, 0.90-1.08). The greatest overestimation was among Chinese patients (slope, 0.73; 95% CI, 0.67-0.80) (Figure 2; eFigure 4 in Supplement 1). Calibration slopes for disaggregated Hispanic subgroups were generally close to and included 1, ranging from 0.95 (95% CI, 0.54-1.36) among Puerto Rican participants to 1.04 (95% CI, 0.95-1.13) among other Hispanic participants (Figure 2; eFigure 5 in Supplement 1).

The predicted to observed risk ratio varied from 1.23 among Japanese participants to 1.54 among Chinese participants in Asian subgroups, and from 1.06 among Hispanic participants to 1.19 among Puerto Rican participants for Hispanic subgroups (eTable 3 in Supplement 1). The differences between observed and predicted risk were statistically significant for all Asian subgroups and Mexican patients.

#### ASCVD

The Harrel C statistics for ASCVD (Table 2) ranged from 0.76 (95% CI, 0.75-0.76) in White participants and 0.76 (95% CI, 0.75-0.78) in Black participants to 0.80 (95% CI, 0.79-0.81) in Asian patients, with calibration slopes varying from 0.86 (95% CI, 0.80-0.92) in Asian patients to 1.21 (95% CI, 1.10-1.33) in Black patients (eFigure 6 in Supplement 1). Among disaggregated Asian subgroups (Table 3), C statistics ranged between 0.75 (95% CI, 0.72-0.77) in Filipino patients to 0.82 (95% CI, 0.80-0.84) in Asian Indian patients. The calibration slopes for ASCVD varied slightly, ranging from 0.71 (95% CI, 0.62-0.80) in Filipino patients to 1.01 (95% CI, .94-1.08) in Asian Indian patients (eFigure 7 in Supplement 1). Among disaggregated Hispanic subgroups, C statistics ranged from 0.78 to 0.80. Calibration slopes included 1 among Hispanic subgroups, varying from 0.98 (95% CI, 0.91-1.05) among Mexican participants to 1.07 (95% CI, 0.65-1.49) among Puerto Rican participants (Table 3; eFigure 8 in Supplement 1).

The predicted risk was comparable to the observed risk for ASCVD (3.7%) for the overall population, for Hispanic individuals (3.5%) and for Asian individuals (eFigure 1 in Supplement 1). The predicted to observed risk ratio varied from 1.06 among Japanese participants to 1.34 among Chinese participants for Asian subgroups (eTable 3 in Supplement 1), and less variation was observed among Hispanic subgroups, ranging from 0.99 among other Hispanic participants to 1.25 among Puerto Rican participants. Differences in observed-vs-predicted ratio were only statistically different for Asian Indian, Chinese, Filipino, and other Asian participants (eTable 3 in Supplement 1).

#### HF

The Harrel C statistics for HF were 0.88 (95% CI, 0.87-0.89) for Asian patients and 0.84 (95% CI, 0.83-0.86) for Hispanic groups (Table 2). Calibration slopes were 0.79 (95% CI, 0.69-0.89) for Asian patients and 0.98 (95% CI, 0.88-1.07) for Hispanic patients (Table 2; eFigure 9 in Supplement 1).

Among disaggregated Asian subgroups (Table 3), C statistics ranged from 0.84 (95% CI, 0.81-0.88) in Japanese patients to 0.90 (95% CI, 0.88-0.93) in Asian Indian patients. C statistics ranged from 0.81 (95% CI, 0.75-0.87) in Puerto Rican patients to 0.85 (95% CI, 0.83-0.86) in Mexican patients among disaggregated Hispanic subgroups. Calibration slopes among disaggregated Asian subgroups ranged from 0.67 (95% CI, 0.56-0.77) in Chinese patients to 0.92 (95% CI, 0.78-1.07) in Asian Indian patients (eFigure 10 in Supplement 1). Calibration curves among Hispanic subgroups included 1 for Puerto Rican patients (slope, 0.73; 95% CI, 0.33-1.13), Mexican patients (slope, 0.95; 95% CI, 0.84-1.07), and other Hispanic patients (slope, 0.99; 95% CI, 0.92-1.07) (eFigure 11 in Supplement 1) (eTable 3 in Supplement 1).

### Comparison to PCE Model

In total, 298 276 patients aged 40 to 79 years were used to compare model performance between PREVENT and PCE. C statistics were similar between the 2 predictive models across racial and ethnic groups and disaggregated subgroups (eTable 1 in Supplement 1). However, the calibration slopes for PCE model were significantly less than 1.0 for all racial and ethnic groups and subgroups, representing substantial overestimation of ASCVD risk. In contrast, calibration slopes for the PREVENT ASCVD equation were closer to 1.0 among all racial and ethnic groups and disaggregated subgroups, with most 95% CIs covering 1.0, implying excellent calibration (eTable 2 in Supplement 1).

## Discussion

To our knowledge, this cohort study is the first evaluation of how the PREVENT equations perform in a large sample of disaggregated Asian and Hispanic subgroups using real world data. This cohort included more than 361 000 community-based patients in which one-third were Asian or Hispanic.

Our results show that PREVENT equations performed well in this study cohort and similarly to the original equation development and validation cohort on the discrimination measure (ie, the Harrel C statistic).^2^ In particular, the performance was slightly better in discriminating CVD events for Asian and Hispanic participants compared to Black or White participants in the study population. The equations slightly overestimated CVD risk for all 3 CVD event types in Asian and most Asian subgroups, and accurately predicted CVD events among Hispanic and disaggregated Hispanic subgroups.

Although the risk of CVD events was well calibrated in this study cohort, noticeable variation was observed by race and ethnicity. Heterogeneity of cardiovascular risk among race and ethnic groups has been widely reported, particularly within Asian^11,12^ and Hispanic subgroups.^7,13,14^ The variation in calibration among racial and ethnic groups suggests recalibration in different racial and ethnic groups may help to minimize the impact of the risk model in decision-making.^15^

We found modest differences in the calibration of PREVENT equations in disaggregated Asian and Hispanic subgroups. Disaggregated Asian subgroups differed in risk factor profiles, such as BMI, smoking, and prevalence of type 2 diabetes; less variation among those risk factors was observed within Hispanic subgroups, which may explain the varying level of calibration within subgroups. Comparing 2 subgroups, Filipino and Mexican individuals, who had similar risk profiles (age, BMI, lipid measurements, SBP, type 2 diabetes, and estimated glomerular filtration rate), all 3 PREVENT equations still overestimated CVD risk for Filipino patients but predicted risk well for Mexican individuals. This suggests that the variation of the risk of CVD events in subgroups goes beyond risk factors included in the PREVENT equation and highlights the importance of considering other individual-level risk factors in combination with risk predicted by the PREVENT equations.

Compared with the PCE, a widely used model for risk stratification to guide clinical decisions in primary ASCVD prevention, our study further confirmed that the PREVENT equations have significantly improved performance.^2,3,16^ Studies consistently showed that PCE overestimated ASCVD, regardless of sex, race, ethnicity, or ASCVD risk group.^7,17^^,^ Our data further demonstrated that the PCE model significantly overestimated the ASCVD risk among all racial and ethnic groups. Compared to prior work^10^ that assessed the PCE performance in disaggregated Asian and Hispanic subgroups, our results reiterate substantially better calibration performance for PREVENT equations among the same disaggregated Asian and Hispanic subgroups, while still maintaining comparable discrimination capacity. To better inform their clinical application, future work might focus on providing clinically sensible cutoffs based on PREVENT ASCVD risk to guide treatment decisions, much like the guideline recommendations based on PCE.^1^

### Strengths and Limitations

Our study has several strengths. First, the inclusion of disaggregated Asian and Hispanic groups provides initial insight into the performance of PREVENT in these heterogeneous groups. Second, our study examined a diverse patient population with 8.1 years of follow-up time, allowing us to validate 10-year risk equations with adequate follow-up time.

Our findings should be interpreted in the context of some limitations. First, despite disaggregation of Asian and Hispanic subgroups, we were unable to fully examine other disaggregated groups (eg, Laotian and Columbian) as well as less populous race groups (eg, American Indian and Alaska Native, Native Hawaiian or Other Pacific Islander, and multiple races). Moreover, comparisons of predictive utility of PREVENT and PCEs across disaggregated Asian and Hispanic subgroups were limited by small sample sizes in some of these subgroups. Second, as a health care system–based study, our population may be biased to include individuals who are less healthy compared to the general population. For example, patients with incomplete data might systematically differ from our study cohort. Our data showed that almost half of eligible patients (n = 339 715) had incomplete data (eTable 4 in Supplement 1) and were excluded from the analysis. To reduce missing data, we restricted the study cohort to the primary care population and incorporated CareEverywhere data, data shared through intersystem health information exchange, to reduce data leakage due to care sought outside of the studied health care system. Using CareEverywhere data, we were able to capture 2% more outcomes. Third, although outcome ascertainment mirrored the PREVENT derivation outcomes, *ICD* diagnostic codes were commonly used to define outcomes. Incorrectly documented diagnosis is common in clinical practice, and chart review is needed to examine the quality of the outcomes and will be included in a future study.

## Conclusions

Using EHR data from a large, diverse health care system, we found that the newly developed PREVENT equations had good discrimination and calibration among diverse populations and among Asian and Hispanic subgroups, with small differences in model performance within these heterogenous populations. These findings further emphasize the generalizability of PREVENT equations, even among racial and ethnic groups that were not well represented in the model development cohort.

## Notes

Supplement 1. eMethods eTable 1. Harrel’s C-statistics comparison between PREVENT 10-year ASCVD equation and 10-year PCE for overall (age 40-79 years), stratified by race/ethnicity and subgroups in Asian and Hispanic (N=298,276) eTable 2. Calibration slope comparison between PREVENT ASCVD equation and PCE for overall (age 40-79 years), stratified by race/ethnicity and subgroups in Asian and Hispanic (N=298,276) eTable 3. Predicted vs. Observed Rates, predicted-to-observed-relative-ratio of total CVD, ASCVD and HF, overall and by Race/Ethnicity and Disaggregated Non-Hispanic Asian and Hispanic subgroups for the study cohort eTable 4. Comparing the patients who were included in the analysis to those with incomplete predictors eFigure 1. Comparison of 10-year observed vs. predicted ASCVD risk by race/ethnicity eFigure 2. Comparison of 10-year observed vs. predicted Heart failure risk by race/ethnicity eFigure 3. Calibration curve for Total CVD, stratified by race/ethnicity group eFigure 4. Calibration curve for Total CVD, stratified by Asian subgroups eFigure 5. Calibration curve for Total CVD, stratified by Hispanic subgroups eFigure 6. Calibration curve for ASCVD, stratified by race/ethnicity group eFigure 7. Calibration curve for ASCVD, stratified by Asian subgroups eFigure 8. Calibration curve for ASCVD, stratified by Hispanic subgroups eFigure 9. Calibration curve for HF, stratified by race/ethnicity group eFigure 10. Calibration curve for HF, stratified by Asian subgroups eFigure 11. Calibration curve for HF, stratified by Hispanic subgroups

Supplement 2. Data sharing statement
