# Supplementary material for: Performance of the American Heart Association’s PREVENT Equations Among Disaggregated Racial and Ethnic Subgroups
Source: JAMA Cardiol. 2025 Jun 25;10(9):876–85. doi: 10.1001/jamacardio.2025.1865 (PMC12199177; doi:10.1001/jamacardio.2025.1865)
Supplement: Supplement 1. — eMethods eTable 1. Harrel’s C-statistics comparison between PREVENT 10-year ASCVD equation and 10-year PCE for overall (age 40-79 years), stratified by race/ethnicity and subgroups in Asian and Hispanic (N=298,276) eTable 2. Calibration slope comparison between PREVENT ASCVD equation and PCE for overall (age 40-79 years), stratified by race/ethnicity and subgroups in Asian and Hispanic (N=298,276) eTable 3. Predicted vs. Observed Rates, predicted-to-observed-relative-ratio of total CVD, ASCVD and HF, overall and by Race/Ethnicity and Disaggregated Non-Hispanic Asian and Hispanic subgroups for the study cohort eTable 4. Comparing the patients who were included in the analysis to those with incomplete predictors eFigure 1. Comparison of 10-year observed vs. predicted ASCVD risk by race/ethnicity eFigure 2. Comparison of 10-year observed vs. predicted Heart failure risk by race/ethnicity eFigure 3. Calibration curve for Total CVD, stratified by race/ethnicity group eFigure 4. Calibration curve for Total CVD, stratified by Asian subgroups eFigure 5. Calibration curve for Total CVD, stratified by Hispanic subgroups eFigure 6. Calibration curve for ASCVD, stratified by race/ethnicity group eFigure 7. Calibration curve for ASCVD, stratified by Asian subgroups eFigure 8. Calibration curve for ASCVD, stratified by Hispanic subgroups eFigure 9. Calibration curve for HF, stratified by race/ethnicity group eFigure 10. Calibration curve for HF, stratified by Asian subgroups eFigure 11. Calibration curve for HF, stratified by Hispanic subgroups [file jamacardiol-e251865-s001.pdf]

Supplemental Online Content

Yan X, Bacong AM, Huang Q, et al. Performance of the American Heart Association's PREVENT equations among disaggregated racial and ethnic subgroups. *JAMA Cardiol*. Published online June 25, 2025. doi:10.1001/jamacardio.2025.1865

eMethods

eTable 1. Harrel's C-statistics comparison between PREVENT 10-year ASCVD equation and 10-year PCE for overall (age 40-79 years), stratified by race/ethnicity and subgroups in Asian and Hispanic (N=298,276)

eTable 2. Calibration slope comparison between PREVENT ASCVD equation and PCE for overall (age 40-79 years), stratified by race/ethnicity and subgroups in Asian and Hispanic (N=298,276)

eTable 3. Predicted vs. Observed Rates, predicted-to-observed-relative-ratio of total CVD, ASCVD and HF, overall and by Race/Ethnicity and Disaggregated Non-Hispanic Asian and Hispanic subgroups for the study cohort

eTable 4. Comparing the patients who were included in the analysis to those with incomplete predictors

eFigure 1. Comparison of 10-year observed vs. predicted ASCVD risk by race/ethnicity

eFigure 2. Comparison of 10-year observed vs. predicted Heart failure risk by race/ethnicity

eFigure 3. Calibration curve for Total CVD, stratified by race/ethnicity group

eFigure 4. Calibration curve for Total CVD, stratified by Asian subgroups

eFigure 5. Calibration curve for Total CVD, stratified by Hispanic subgroups

eFigure 6. Calibration curve for ASCVD, stratified by race/ethnicity group

eFigure 7. Calibration curve for ASCVD, stratified by Asian subgroups

© 2025 Yan X et al. *JAMA Cardiology*.

eFigure 8. Calibration curve for ASCVD, stratified by Hispanic subgroups

eFigure 9. Calibration curve for HF, stratified by race/ethnicity group

eFigure 10. Calibration curve for HF, stratified by Asian subgroups

eFigure 11. Calibration curve for HF, stratified by Hispanic subgroups

This supplemental material has been provided by the authors to give readers additional information about their work.

© 2025 Yan X et al. *JAMA Cardiology*.

eMethods.

eMethods 1.1 Definitions for "Other Asian" and "Other Hispanic"

We categorize patients as "Other Asian" based on following operational rules:

1. Patient reported "Non-Hispanic" in the ethnicity, and
2. Patient reported region of origin as "Other Asian".
   Or
   Patients report multiple regions of origin, and at least one is Asian country/region. Detailed multi-racial combination is shown below.

Asian Category	Ethnicity	RACE1	RACE2
Other Asian	NON-HISPANIC	AMERICAN INDIAN OR ALASKA NATIVE	OTHER ASIAN
Other Asian	NON-HISPANIC	ASIAN INDIAN	CHINESE
Other Asian	NON-HISPANIC	ASIAN INDIAN	FILIPINO
Other Asian	NON-HISPANIC	ASIAN INDIAN	JAPANESE
Other Asian	NON-HISPANIC	ASIAN INDIAN	KOREAN
Other Asian	NON-HISPANIC	ASIAN INDIAN	VIETNAMESE
Other Asian	NON-HISPANIC	CHINESE	ASIAN INDIAN
Other Asian	NON-HISPANIC	CHINESE	FILIPINO
Other Asian	NON-HISPANIC	CHINESE	JAPANESE
Other Asian	NON-HISPANIC	CHINESE	KOREAN
Other Asian	NON-HISPANIC	CHINESE	VIETNAMESE
Other Asian	NON-HISPANIC	FILIPINO	ASIAN INDIAN
Other Asian	NON-HISPANIC	FILIPINO	CHINESE
Other Asian	NON-HISPANIC	FILIPINO	JAPANESE
Other Asian	NON-HISPANIC	FILIPINO	KOREAN
Other Asian	NON-HISPANIC	FILIPINO	VIETNAMESE
Other Asian	NON-HISPANIC	JAPANESE	CHINESE
Other Asian	NON-HISPANIC	JAPANESE	FILIPINO
Other Asian	NON-HISPANIC	JAPANESE	KOREAN
Other Asian	NON-HISPANIC	KOREAN	ASIAN INDIAN

© 2025 Yan X et al. *JAMA Cardiology*.

Asian Category	Ethnicity	RACE1	RACE2
Other Asian	NON-HISPANIC	KOREAN	JAPANESE
Other Asian	NON-HISPANIC	KOREAN	VIETNAMESE
Other Asian	NON-HISPANIC	NATIVE HAWAIIAN	OTHER ASIAN
Other Asian	NON-HISPANIC	OTHER ASIAN	
Other Asian	NON-HISPANIC	OTHER ASIAN	AMERICAN INDIAN OR ALASKA NATIVE
Other Asian	NON-HISPANIC	OTHER ASIAN	GUAMANIAN OR CHAMORRO
Other Asian	NON-HISPANIC	OTHER ASIAN	NATIVE HAWAIIAN
Other Asian	NON-HISPANIC	OTHER ASIAN	OTHER
Other Asian	NON-HISPANIC	OTHER ASIAN	OTHER ASIAN
Other Asian	NON-HISPANIC	OTHER ASIAN	OTHER PACIFIC ISLANDER
Other Asian	NON-HISPANIC	OTHER ASIAN	WHITE/CAUCASIAN
Other Asian	NON-HISPANIC	OTHER PACIFIC ISLANDER	OTHER ASIAN
Other Asian	NON-HISPANIC	SAMOAN	OTHER ASIAN
Other Asian	NON-HISPANIC	VIETNAMESE	CHINESE
Other Asian	NON-HISPANIC	VIETNAMESE	KOREAN
Other Asian	NON-HISPANIC	WHITE/CAUCASIAN	OTHER ASIAN

“Other Hispanic” is defined as patient reported their ethnicity is Hispanic regardless race group, but region is not in Mexico, Puerto Rico. The next most populated ethnicity is “Cuban”, however, the size is smaller than 200 in the final analysis cohort, therefore it has been merged into “Other Hispanic”. Please note that for patients who reported as Other Hispanic, their self-reported race can be race category. Following table shows the combination of ethnicity and race groups that have been recategorized as “Other Hispanic”:

© 2025 Yan X et al. *JAMA Cardiology*.

ETHNICITY	RACE1	RACE2
CUBAN	AMERICAN INDIAN OR ALASKA NATIVE	BLACK/AFRICAN AMERICAN
CUBAN	AMERICAN INDIAN OR ALASKA NATIVE	OTHER PACIFIC ISLANDER
CUBAN	BLACK/AFRICAN AMERICAN	
CUBAN	BLACK/AFRICAN AMERICAN	CHINESE
CUBAN	OTHER	
CUBAN	OTHER PACIFIC ISLANDER	
CUBAN	PREFER NOT TO ANSWER	
CUBAN	UNKNOWN	
CUBAN	WHITE/CAUCASIAN	
OTHER HISPANIC/LATINO/SPANISH ORIGIN	AMERICAN INDIAN OR ALASKA NATIVE	
OTHER HISPANIC/LATINO/SPANISH ORIGIN	AMERICAN INDIAN OR ALASKA NATIVE	BLACK/AFRICAN AMERICAN
OTHER HISPANIC/LATINO/SPANISH ORIGIN	AMERICAN INDIAN OR ALASKA NATIVE	CHINESE
OTHER HISPANIC/LATINO/SPANISH ORIGIN	AMERICAN INDIAN OR ALASKA NATIVE	FILIPINO
OTHER HISPANIC/LATINO/SPANISH ORIGIN	AMERICAN INDIAN OR ALASKA NATIVE	JAPANESE

© 2025 Yan X et al. *JAMA Cardiology*.

ETHNICITY	RACE1	RACE2
OTHER HISPANIC/LATINO/SPANISH ORIGIN	AMERICAN INDIAN OR ALASKA NATIVE	OTHER ASIAN
OTHER HISPANIC/LATINO/SPANISH ORIGIN	AMERICAN INDIAN OR ALASKA NATIVE	WHITE/CAUCASIAN
OTHER HISPANIC/LATINO/SPANISH ORIGIN	ASIAN INDIAN	
OTHER HISPANIC/LATINO/SPANISH ORIGIN	ASIAN INDIAN	FILIPINO
OTHER HISPANIC/LATINO/SPANISH ORIGIN	ASIAN INDIAN	OTHER ASIAN
OTHER HISPANIC/LATINO/SPANISH ORIGIN	ASIAN INDIAN	WHITE/CAUCASIAN
OTHER HISPANIC/LATINO/SPANISH ORIGIN	BLACK/AFRICAN AMERICAN	
OTHER HISPANIC/LATINO/SPANISH ORIGIN	BLACK/AFRICAN AMERICAN	AMERICAN INDIAN OR ALASKA NATIVE
OTHER HISPANIC/LATINO/SPANISH ORIGIN	BLACK/AFRICAN AMERICAN	FILIPINO

© 2025 Yan X et al. *JAMA Cardiology*.

ETHNICITY	RACE1	RACE2
OTHER HISPANIC/LATINO/SPANISH ORIGIN	BLACK/AFRICAN AMERICAN	WHITE/CAUCASIAN
OTHER HISPANIC/LATINO/SPANISH ORIGIN	CHINESE	
OTHER HISPANIC/LATINO/SPANISH ORIGIN	CHINESE	FILIPINO
OTHER HISPANIC/LATINO/SPANISH ORIGIN	CHINESE	KOREAN
OTHER HISPANIC/LATINO/SPANISH ORIGIN	CHINESE	OTHER
OTHER HISPANIC/LATINO/SPANISH ORIGIN	CHINESE	OTHER ASIAN
OTHER HISPANIC/LATINO/SPANISH ORIGIN	CHINESE	PREFER NOT TO ANSWER
OTHER HISPANIC/LATINO/SPANISH ORIGIN	FILIPINO	
OTHER HISPANIC/LATINO/SPANISH ORIGIN	FILIPINO	CHINESE

© 2025 Yan X et al. *JAMA Cardiology*.

ETHNICITY	RACE1	RACE2
OTHER HISPANIC/LATINO/SPANISH ORIGIN	FILIPINO	NATIVE HAWAIIAN
OTHER HISPANIC/LATINO/SPANISH ORIGIN	FILIPINO	OTHER ASIAN
OTHER HISPANIC/LATINO/SPANISH ORIGIN	FILIPINO	OTHER PACIFIC ISLANDER
OTHER HISPANIC/LATINO/SPANISH ORIGIN	FILIPINO	WHITE/CAUCASIAN
OTHER HISPANIC/LATINO/SPANISH ORIGIN	GUAMANIAN OR CHAMORRO	
OTHER HISPANIC/LATINO/SPANISH ORIGIN	GUAMANIAN OR CHAMORRO	WHITE/CAUCASIAN
OTHER HISPANIC/LATINO/SPANISH ORIGIN	JAPANESE	
OTHER HISPANIC/LATINO/SPANISH ORIGIN	JAPANESE	WHITE/CAUCASIAN
OTHER HISPANIC/LATINO/SPANISH ORIGIN	KOREAN	

© 2025 Yan X et al. *JAMA Cardiology*.

ETHNICITY	RACE1	RACE2
OTHER HISPANIC/LATINO/SPANISH ORIGIN	NATIVE HAWAIIAN	
OTHER HISPANIC/LATINO/SPANISH ORIGIN	NATIVE HAWAIIAN	WHITE/CAUCASIAN
OTHER HISPANIC/LATINO/SPANISH ORIGIN	OTHER	
OTHER HISPANIC/LATINO/SPANISH ORIGIN	OTHER	OTHER
OTHER HISPANIC/LATINO/SPANISH ORIGIN	OTHER	PREFER NOT TO ANSWER
OTHER HISPANIC/LATINO/SPANISH ORIGIN	OTHER	UNKNOWN
OTHER HISPANIC/LATINO/SPANISH ORIGIN	OTHER ASIAN	
OTHER HISPANIC/LATINO/SPANISH ORIGIN	OTHER ASIAN	NATIVE HAWAIIAN
OTHER HISPANIC/LATINO/SPANISH ORIGIN	OTHER ASIAN	OTHER PACIFIC ISLANDER

© 2025 Yan X et al. *JAMA Cardiology*.

ETHNICITY	RACE1	RACE2
OTHER HISPANIC/LATINO/SPANISH ORIGIN	OTHER ASIAN	WHITE/CAUCASIAN
OTHER HISPANIC/LATINO/SPANISH ORIGIN	OTHER PACIFIC ISLANDER	
OTHER HISPANIC/LATINO/SPANISH ORIGIN	OTHER PACIFIC ISLANDER	WHITE/CAUCASIAN
OTHER HISPANIC/LATINO/SPANISH ORIGIN	PREFER NOT TO ANSWER	
OTHER HISPANIC/LATINO/SPANISH ORIGIN	PREFER NOT TO ANSWER	PREFER NOT TO ANSWER
OTHER HISPANIC/LATINO/SPANISH ORIGIN	PREFER NOT TO ANSWER	UNKNOWN
OTHER HISPANIC/LATINO/SPANISH ORIGIN	SAMOAN	
OTHER HISPANIC/LATINO/SPANISH ORIGIN	SAMOAN	NATIVE HAWAIIAN
OTHER HISPANIC/LATINO/SPANISH ORIGIN	UNKNOWN	

© 2025 Yan X et al. *JAMA Cardiology*.

ETHNICITY	RACE1	RACE2
OTHER HISPANIC/LATINO/SPANISH ORIGIN	VIETNAMESE	
OTHER HISPANIC/LATINO/SPANISH ORIGIN	VIETNAMESE	OTHER ASIAN
OTHER HISPANIC/LATINO/SPANISH ORIGIN	WHITE/CAUCASIAN	
OTHER HISPANIC/LATINO/SPANISH ORIGIN	WHITE/CAUCASIAN	AMERICAN INDIAN OR ALASKA NATIVE
OTHER HISPANIC/LATINO/SPANISH ORIGIN	WHITE/CAUCASIAN	BLACK/AFRICAN AMERICAN
OTHER HISPANIC/LATINO/SPANISH ORIGIN	WHITE/CAUCASIAN	CHINESE
OTHER HISPANIC/LATINO/SPANISH ORIGIN	WHITE/CAUCASIAN	FILIPINO
OTHER HISPANIC/LATINO/SPANISH ORIGIN	WHITE/CAUCASIAN	GUAMANIAN OR CHAMORRO
OTHER HISPANIC/LATINO/SPANISH ORIGIN	WHITE/CAUCASIAN	JAPANESE

© 2025 Yan X et al. *JAMA Cardiology*.

ETHNICITY	RACE1	RACE2
OTHER HISPANIC/LATINO/SPANISH ORIGIN	WHITE/CAUCASIAN	KOREAN
OTHER HISPANIC/LATINO/SPANISH ORIGIN	WHITE/CAUCASIAN	NATIVE HAWAIIAN
OTHER HISPANIC/LATINO/SPANISH ORIGIN	WHITE/CAUCASIAN	OTHER
OTHER HISPANIC/LATINO/SPANISH ORIGIN	WHITE/CAUCASIAN	OTHER PACIFIC ISLANDER
OTHER HISPANIC/LATINO/SPANISH ORIGIN	WHITE/CAUCASIAN	PREFER NOT TO ANSWER

© 2025 Yan X et al. *JAMA Cardiology*.

eMethods 1.2 ICD, CPT codes and medications for predictors and outcomes.

ICD codes for outcomes[a]

PREVENT Outcome	Outcome	ICD-9 codes	ICD-10 codes
ASCVD	Myocardial infarction (MI)	410	I21, I22
	Hemorrhagic and Ischemic stroke	431, 432, 433.x1, 434.x1	I61, I62, I63
Heart Failure	Heart Failure	428	I50

[a]Adopted codes from Khan SS, Matsushita K, Sang Y, Ballew SH, Grams ME, Surapaneni A, Blaha MJ, Carson AP, Chang AR, Ciemins E, Go AS, Gutierrez OM, Hwang SJ, Jassal SK, Kovesdy CP, Lloyd-Jones DM, Shlipak MG, Palaniappan LP, Sperling L, Virani SS, Tuttle K, Neeland IJ, Chow SL, Rangaswami J, Pencina MJ, Ndumele CE, Coresh J; Chronic Kidney Disease Prognosis Consortium and the American Heart Association Cardiovascular-Kidney-Metabolic Science Advisory Group. Development and Validation of the American Heart Association's PREVENT Equations. Circulation. 2024 Feb 6;149(6):430-449. doi: 10.1161/CIRCULATIONAHA.123.067626. Epub 2023 Nov 10. Erratum in: Circulation. 2024 Mar 12;149(11):e956. doi: 10.1161/CIR.0000000000001230. PMID: 37947085; PMCID: PMC10910659.

ICD/CPT codes for predictors

Predictors	ICD or CPT or Medications	Codes	Note
HDL/Total cholesterol	CPT	83718/82465	
eGFR (creatinine)	CPT	82565	
HbA1c	CPT	83036	
Diabetes	ICD-9/ICD-10	ICD-9 codes:<br>250.01, 250.03, 250.11, 250.13, 250.21, 250.23, 250.31, 250.33, 250.41, 250.43, 250.51, 250.53, 250.61, 250.63, 250.8,1 250.83, 250.91, 250.93 250.00, 250.02, 250.10 250.12, 250.20, 250.22 250.30, 250.32, 250.40 250.42, 250.50, 250.52 250.60, 250.62, 250.70 250.72, 250.80, 250.82 250.90, 250.92, 357.2, 362.0, 366.41<br><br>ICD-10 codes:<br>E10	Criteria:<br><br>2+ ICD codes on different date (>=1 day)

© 2025 Yan X et al. *JAMA Cardiology*.

Predictors	ICD or CPT or Medications	Codes	Note
		E11<br>E13.42<br>E13.59<br>E13.65, E13.69<br>E13.8<br>E13.9<br>E13.00, E13.01<br>E13.10, E13.11, E13.21<br>E13.22, E13.29<br>E13.35, E13.36<br>E13.42<br>E13.52<br>E13.610, E13.618<br>E13.620, E13.621, E13.622<br>E13.628<br>E13.630, E13.638<br>E13.641, E13.649<br>E13.3, E13.4, E13.5, E13.6<br>Z79.84<br>Z79.4	
Hypertension	ICD-9/ICD-10	ICD-9 codes:<br>401-405<br><br>ICD-10 codes:<br>I10<br>I11.0, I11.9<br>I12.0, I12.9<br>I13.0, I13.10, I13.11<br>I13.2<br>I15.0, I15.1, I15.2, I15.8<br>I15.9<br>I16.0, I16.1, I16.9	Criteria:<br><br>2+ ICD codes on different date (>=1 day)
Anti-hypertensive medication	Medication Class	ACE Inhibitors, ARBs, Alpha Blockers, Beta Blockers, Calcium Channel Blockers, Diuretics	Use medication class in the medication order data
Statin	Medication Type	Lovastatin, Simvastatin, Pravastatin, Fluvastatin, Atorvastatin, and Rosuvastatin	Use medication class in the medication order data

© 2025 Yan X et al. *JAMA Cardiology*.

**Predictors**	**ICD or CPT or Medications**	**Codes**	**Note**
Current Smoking	No code	From Health Behavior Table	
BMI	No Code	From Encounter Vital Data	Use the one prior to and closest to the index date
SBP	No Code	From Encounter Vital Data	Use the one prior to and closest to the index date

© 2025 Yan X et al. *JAMA Cardiology*.

eTable 1. Harrel's C-statistics comparison between PREVENT 10-year ASCVD equation and 10-year PCE for overall (age 40-79 years), stratified by race/ethnicity and subgroups in Asian and Hispanic (N=298,276).

	**PREVENT ASCVD Equation**		**PCE Model**	
Overall	0.75 (0.74-0.76)		0.75 (0.74-0.76)	
	Female	Male	Female	Male
White	0.75 (0.74, 0.76)	0.72 (0.72, 0.73)	0.75 (0.74, 0.76)	0.72 (0.71, 0.73)
Black	0.75 (0.72, 0.78)	0.74 (0.71, 0.77)	0.74 (0.71, 0.77)	0.73 (0.70, 0.76)
Hispanic	0.77 (0.75, 0.79)	0.74 (0.72, 0.76)	0.76 (0.74, 0.78)	0.73 (0.71, 0.75)
Asian	0.78 (0.76, 0.79)	0.74 (0.73, 0.76)	0.77 (0.75, 0.79)	0.74 (0.72, 0.75)
Other	0.78 (0.75, 0.82)	0.73 (0.70, 0.76)	0.77 (0.74, 0.80)	0.72 (0.69, 0.75)
Mexican	0.77 (0.74, 079)	0.73 (0.70, 0.75)	0.76 (0.73, 0.79)	0.72 (0.70, 0.75)
Puerto Rican	0.73 (0.54, 0.92)	0.80 (0.66, 0.93)	0.74 (0.56, 0.93)	0.78 (0.64, 0.91)
Other Hispanic	0.77 (0.75, 0.80)	0.74 (0.72, 0.77)	0.77 (0.74, 0.79)	0.74 (0.71, 0.77)
Asian Indian	0.78 (0.73, 0.82)	0.74 (0.71, 0.78)	0.77 (0.73, 0.82)	0.75 (0.71, 0.78)
Chinese	0.80 (0.77, 0.83)	0.74 (0.71, 0.77)	0.80 (0.77, 0.83)	0.74 (0.71, 0.76)
Filipino	0.71 (0.67, 0.75)	0.72 (0.67, 0.76)	0.71 (0.67, 0.75)	0.71 (0.67, 0.75)
Japanese	0.75 (0.70, 0.80)	0.74 (0.69, 0.79)	0.75 (0.69, 0.80)	0.74 (0.69, 0.80)
Korean	0.75 (0.65, 0.85)	0.81 (0.71, 0.90)	0.75 (0.65, 0.85)	0.80 (0.71, 0.89)
Vietnamese	0.76 (0.63, 0.88)	0.74 (0.65, 0.84)	0.76 (0.64, 0.88)	0.75 (0.65, 0.85)
Other Asian	0.81 (0.77, 0.84)	0.74 (0.70, 0.79)	0.80 (0.76, 0.84)	0.73 (0.68, 0.77)

© 2025 Yan X et al. *JAMA Cardiology*.

eTable 2. Calibration slope comparison between PREVENT ASCVD equation and PCE for overall (age 40-79 years), stratified by race/ethnicity and subgroups in Asian and Hispanic (N=298,276).

	**PREVENT ASCVD Equation**		**PCE Model**	
Overall	1.05 (1.01-1.09)		0.42 (0.38-0.45)	
	Female	Male	Female	Male
White	1.11 (1.04, 1.18)	1.08 (1.04, 1.13)	0.47 (0.39, 0.54)	0.43 (0.41, 0.46)
Black	1.29 (1.14, 1.45)	1.20 (1.03, 1.36)	0.54 (0.48, 0.60)	0.51 (0.46, 0.57)
Hispanic	1.03 (0.95, 1.11)	1.06 (0.97, 1.15)	0.43 (0.35, 0.51)	0.42 (0.38, 0.47)
Asian	0.79 (0.69, 0.89)	0.91 (0.85, 0.98)	0.32 (0.28, 0.36)	0.34 (0.31, 0.37)
Other	0.98 (0.76, 1.19)	1.06 (0.92, 1.19)	0.41 (0.36, 0.47)	0.41 (0.33, 0.51)
Mexican	0.93 (0.79, 1.07)	1.06 (0.98, 1.14)	0.40 (0.33, 0.46)	0.41 (0.35, 0.47)
Puerto Rican	0.82 (0.46, 1.19)	1.66 (0.79, 2.52)	0.41 (0.28, 0.54)	0.59 (0.41, 0.78)
Other Hispanic	1.14 (1.00, 1.28)	1.05 (0.92, 1.17)	0.47 (0.38, 0.55)	0.44 (0.38, 0.49)
Asian Indian	0.84 (0.71, 0.98)	1.09 (0.97, 1.21)	0.37 (0.32, 0.41)	0.41 (0.32, 0.50)
Chinese	0.77 (0.69, 0.85)	0.79 (0.73, 0.84)	0.34 (0.30, 0.38)	0.31 (0.29, 0.34)
Filipino	0.63 (0.56, 0.70)	0.83 (0.65, 1.02)	0.28 (0.22, 0.33)	0.34 (0.28, 0.39)
Japanese	0.91 (0.68, 1.14)	1.03 (0.81, 1.24)	0.37 (0.31, 0.43)	0.41 (0.31, 0.52)
Korean	0.78 (0.46, 1.10)	1.37 (0.87, 1.87)	0.30 (0.16, 0.44)	0.46 (0.34, 0.59)
Vietnamese	0.90 (0.56, 1.24)	0.79 (0.25, 1.34)	0.39 (0.25, 0.53)	0.32 (0.06, 0.58)
Other Asian	0.79 (0.71, 0.86)	0.92 (0.74, 1.11)	0.35 (0.28, 0.41)	0.34 (0.27, 0.41)

Note. C-Statistics are listed with 95% Confidence Intervals.

© 2025 Yan X et al. *JAMA Cardiology*.

eTable 3. Predicted vs. Observed Rates, predicted-to-observed-relative-ratio of total CVD, ASCVD and HF, overall and by Race/Ethnicity and Disaggregated Non-Hispanic Asian and Hispanic subgroups for the study cohort

	n	10-year predicted CVD, %	10-year Observed CVD, %	Predicted-to-Observed Ratio	P value[a]	10-year predicted ASCVD, %	10-year Observed ASCVD, %	Predicted-to-Observed Ratio	P value[a]	10-year predicted HF, %	10-year Observed HF, %	Predicted-to-Observed Ratio	P value[a]
All	361,778	6.29	6.23	1.01	0.11	3.74	3.67	1.02	0.02	3.66	3.5	1.07	<.0001
NHW	193,337	7.34	7.88	0.93	<.0001	4.34	4.48	0.97	0.002	4.37	4.62	0.95	<.0001
NHB	12,134	7.38	9.04	0.82	<.0001	4.27	5.18	0.83	<.0001	4.66	5.59	0.83	<.0001
Hispanic	40,897	5.81	5.51	1.05	0.006	3.50	3.47	1.01	0.69	3.31	2.84	1.17	<.0001
NHA	81,424	4.32	3.18	1.36	<.0001	2.60	2.15	1.21	<.0001	2.31	1.42	1.63	<.0001
NH-Other	11,600	6.35	6.53	0.97	0.39	3.80	3.78	1.00	0.94	3.69	3.90	0.95	0.23
Unknown	22,386	4.63	2.67	1.73	<.0001	2.82	1.65	1.71	<.0001	2.54	1.29	1.98	<.0001
Asian Indian	25,182	2.94	2.30	1.28	<.0001	1.84	1.63	1.13	0.008	1.40	0.94	1.50	<.0001
Chinese	24,313	4.60	2.99	1.54	<.0001	2.74	2.04	1.34	<.0001	2.48	1.30	1.90	<.0001
Filipino	11,539	5.89	4.63	1.27	<.0001	3.49	2.86	1.22	<.0001	3.34	2.41	1.39	<.0001
Japanese	4,174	6.90	5.61	1.23	0.0002	4.04	3.81	1.06	0.43	4.09	2.42	1.69	<.0001
Korean	2,369	4.16	3.04	1.37	0.001	2.49	2.28	1.09	0.48	2.24	1.14	1.96	<.0001
Vietnamese	2,369	3.86	2.91	1.32	0.005	2.33	2.07	1.13	0.36	1.99	1.10	1.81	<.0001
Other Asian	11,478	4.39	3.26	1.35	<.0001	2.64	2.17	1.22	0.0005	2.37	1.51	1.57	<.0001
Mexican	19,528	5.82	5.49	1.06	0.03	3.52	3.46	1.02	0.63	3.31	2.76	1.20	<.0001
Puerto Rican	861	6.10	5.11	1.19	0.17	3.64	2.90	1.25	0.18	3.53	2.90	1.22	0.27
Other Hispanic	20,508	5.79	5.55	1.04	0.12	3.48	3.50	0.99	0.88	3.31	2.91	1.14	0.0004

© 2025 Yan X et al. *JAMA Cardiology*.

eTable 4. Comparing the patients who were included in the analysis to those with incomplete predictors

	**Included in the risk prediction (N= 361,778)**		**Patients with incomplete data [a](N=339,715)**	
**Category N (unless otherwise specified)**	**Female**	**Male**	**Female**	**Male**
**N**	**191,151**	**170,627**	**184,860**	**111,695**
Age, years, mean±SD	53.6±12.8	52.3±12.4	45.8±12.1	46.3±11.9
Race/Ethnicity, %				
Non-Hispanic White	53.3	53.6	53.5	54.3
Non-Hispanic Black	3.6	3.1	3.1	2.8
Hispanic	11.9	10.6	11.3	10.4
Non-Hispanic Asian	22.5	22.5	22.3	21.2
Other or missing	8.7	10.1	9.7	11.3
N of PCP visit in 1-year follow up (mean±SD)	2.6±2.8	1.7±1.9	2.1±2.9	1.2±1.6
% with cardiologist visit in 1-year follow up	5.0	5.9	2	2.5
Diabetes,%	9.5	11.2	1.4	2.1
Current smoking, %	5.1	8.3	5.4	9.9
Antihypertensive treatment, %	34.6	36.3	11.5	11.6
Statin treatment, %	22.1	27.8	2.7	5.9
Death in 10-year follow up, %	1.9	2.2	1.1	1.6

[a] 43,160 missing sex.

© 2025 Yan X et al. *JAMA Cardiology*.

eFigure 1. Comparison of 10-year observed vs. predicted ASCVD risk by race/ethnicity

(A) Overall population by major race/ethnic subgroups, (B) Asian subgroups, (C) Hispanic subgroups.

A

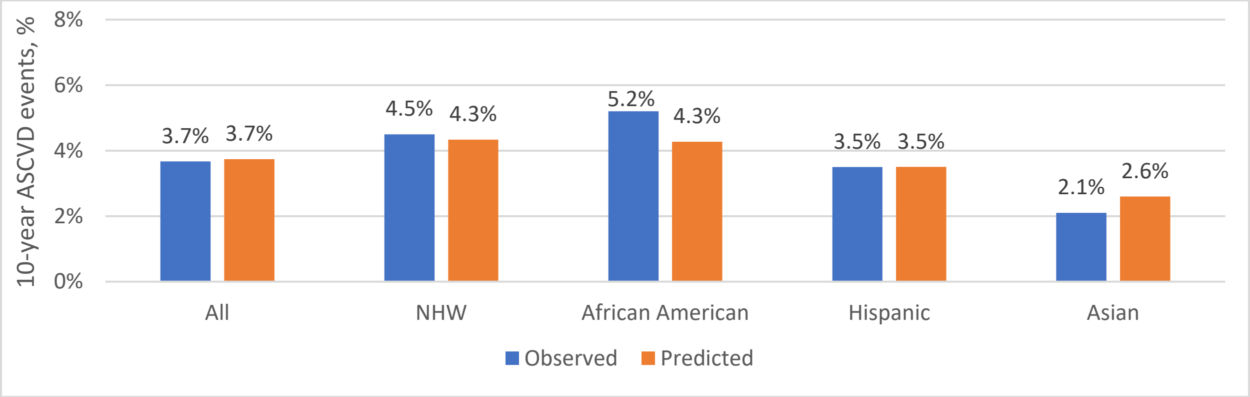

B

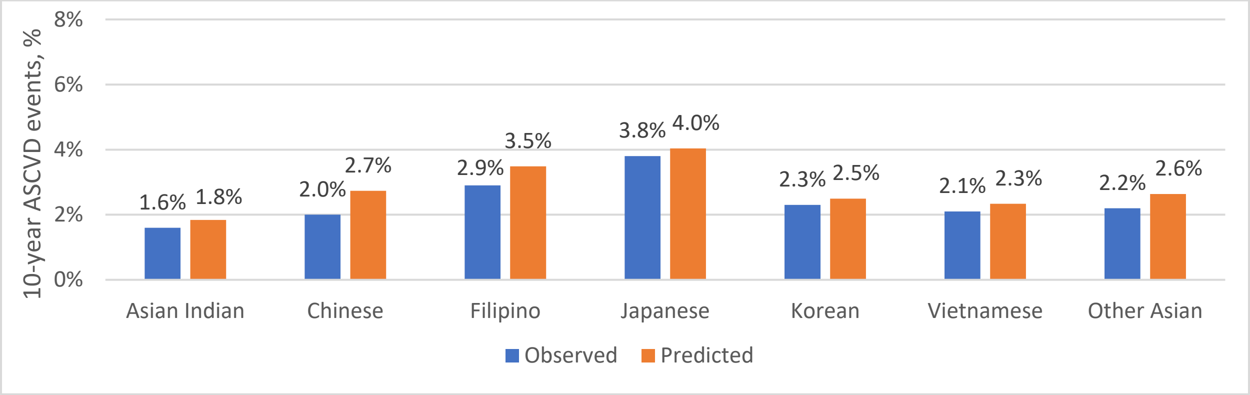

C

© 2025 Yan X et al. *JAMA Cardiology*.

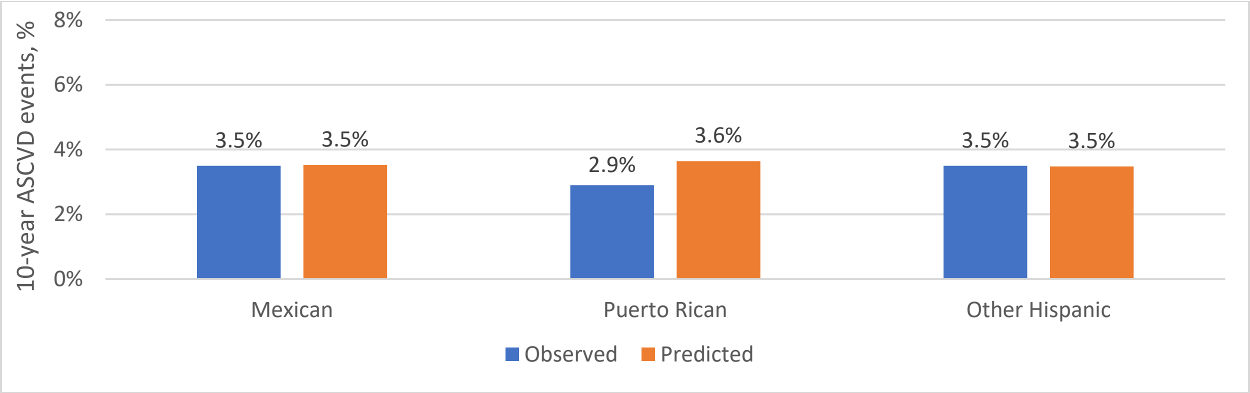

© 2025 Yan X et al. *JAMA Cardiology*.

eFigure 2. Comparison of 10-year observed vs. predicted Heart failure risk by race/ethnicity.
(A) Overall population by major race/ethnic subgroups, (B) Asian subgroups, (C) Hispanic subgroups.

A

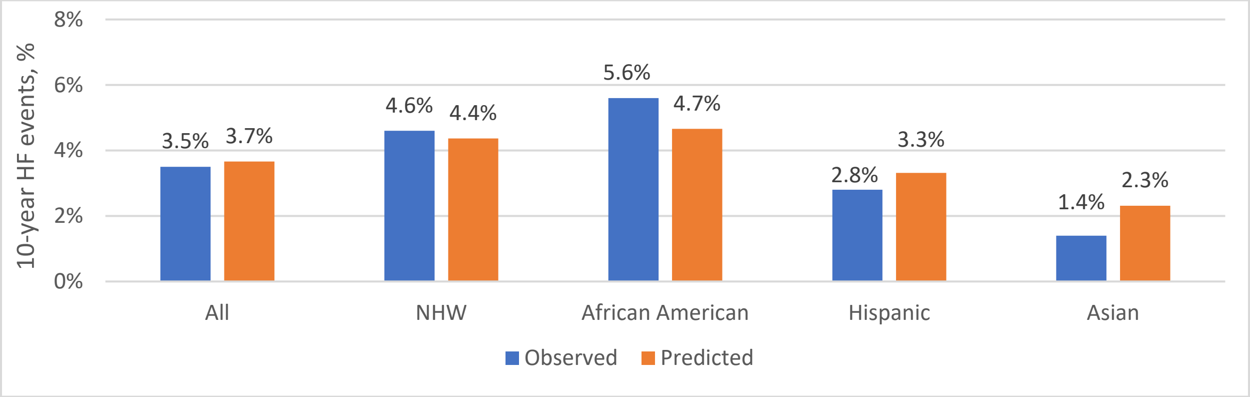

B

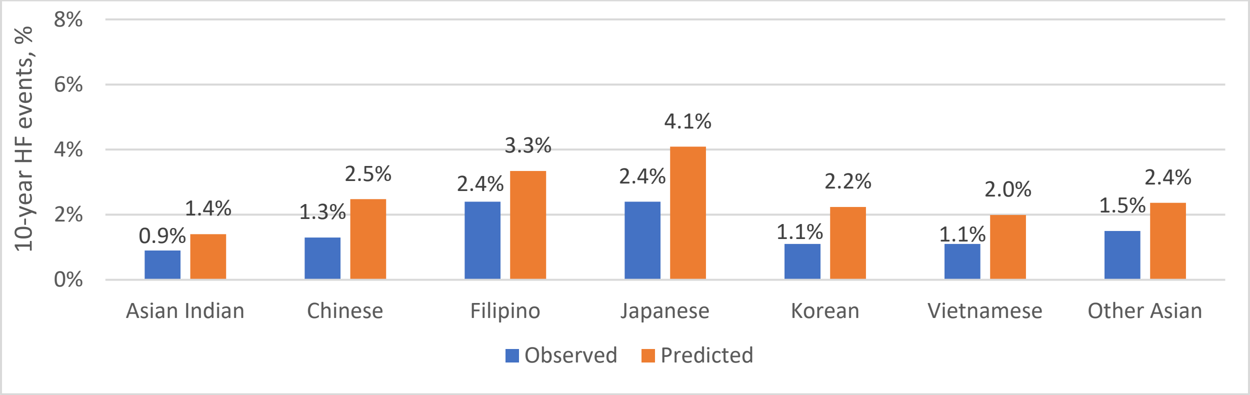

© 2025 Yan X et al. *JAMA Cardiology*.

C

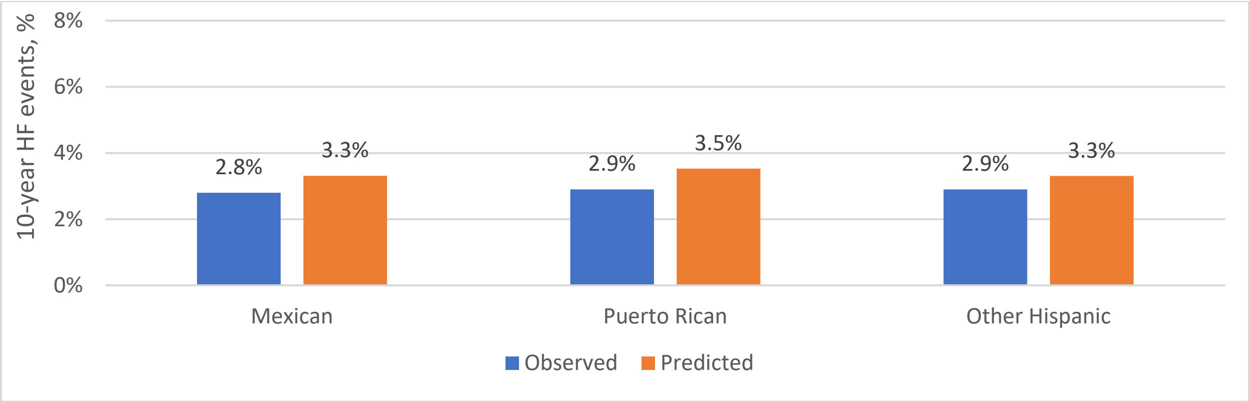

© 2025 Yan X et al. *JAMA Cardiology*.

eFigure 3. Calibration curve for Total CVD, stratified by race/ethnicity group

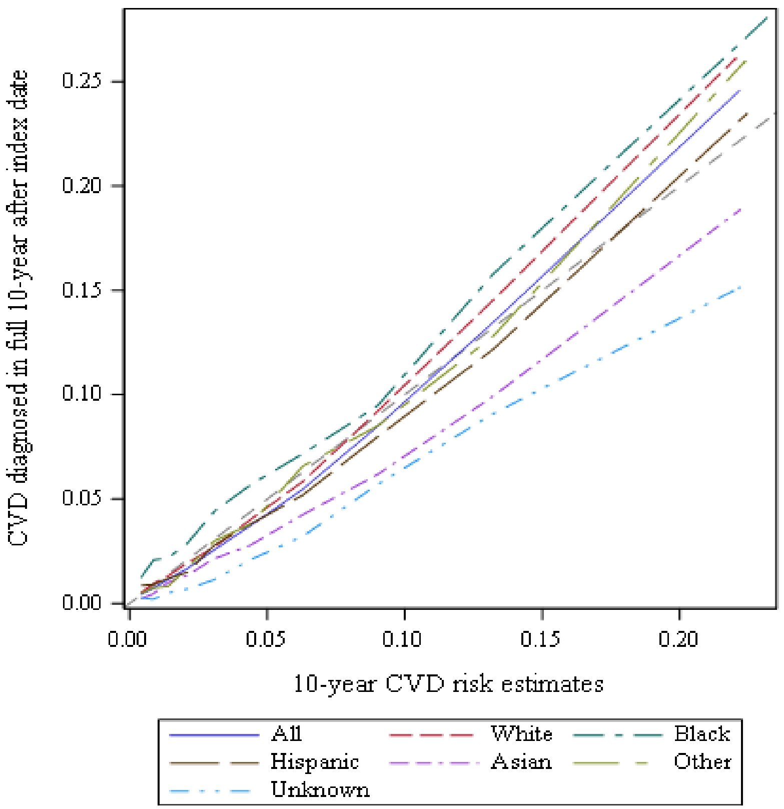

eFigure 4. Calibration curve for Total CVD, stratified by Asian subgroups

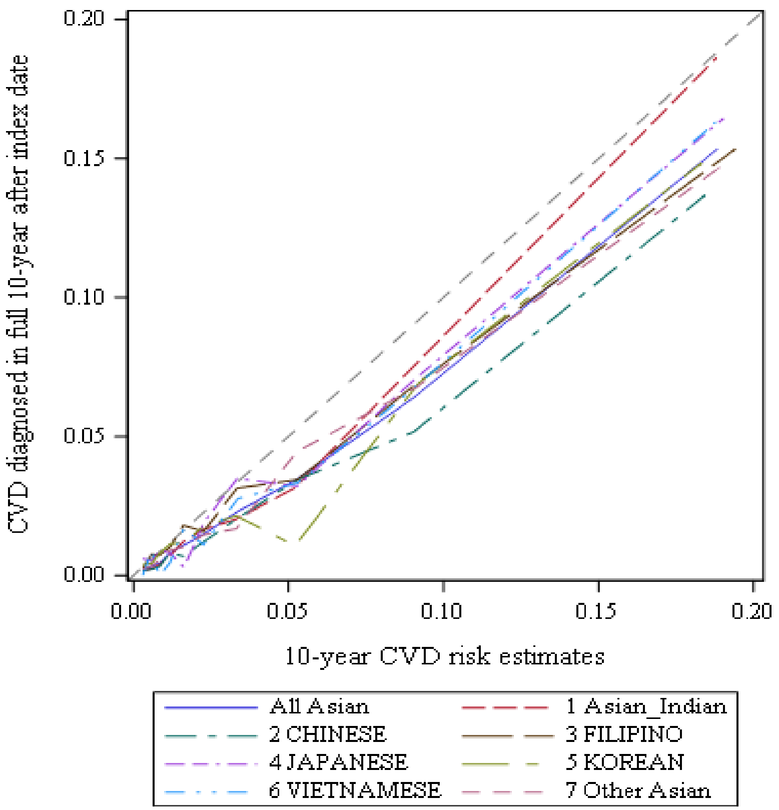

© 2025 Yan X et al. *JAMA Cardiology*.

eFigure 5. Calibration curve for Total CVD, stratified by Hispanic subgroups

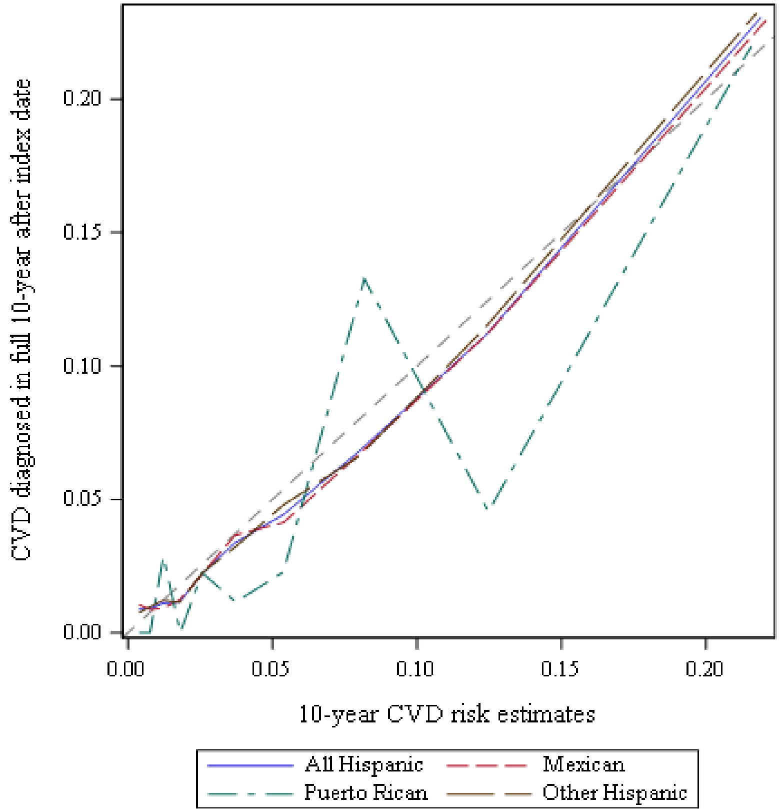

eFigure 6. Calibration curve for ASCVD, stratified by race/ethnicity group

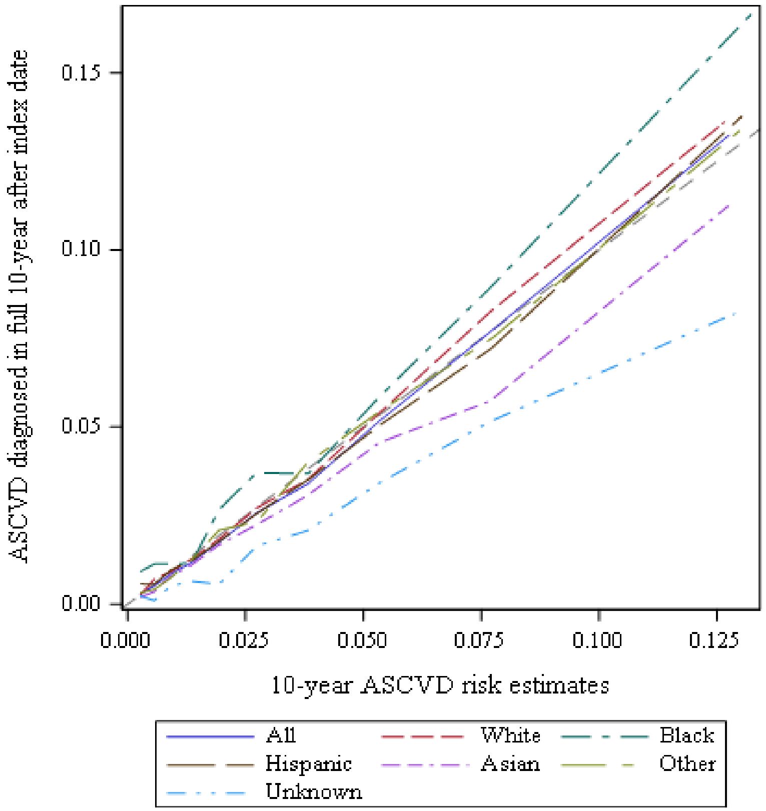

© 2025 Yan X et al. *JAMA Cardiology*.

eFigure 7. Calibration curve for ASCVD, stratified by Asian subgroups

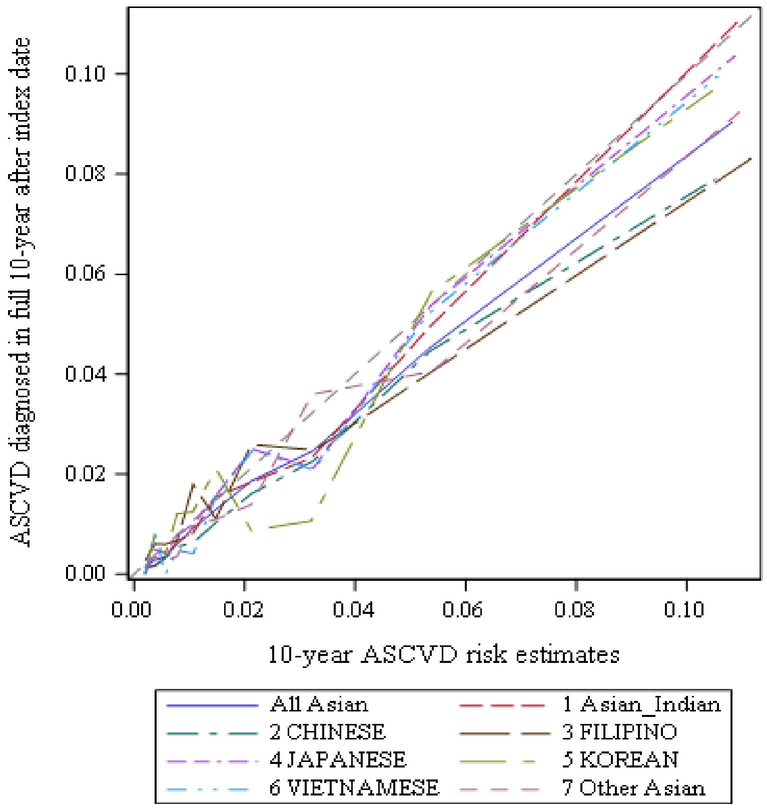

eFigure 8. Calibration curve for ASCVD, stratified by Hispanic subgroups

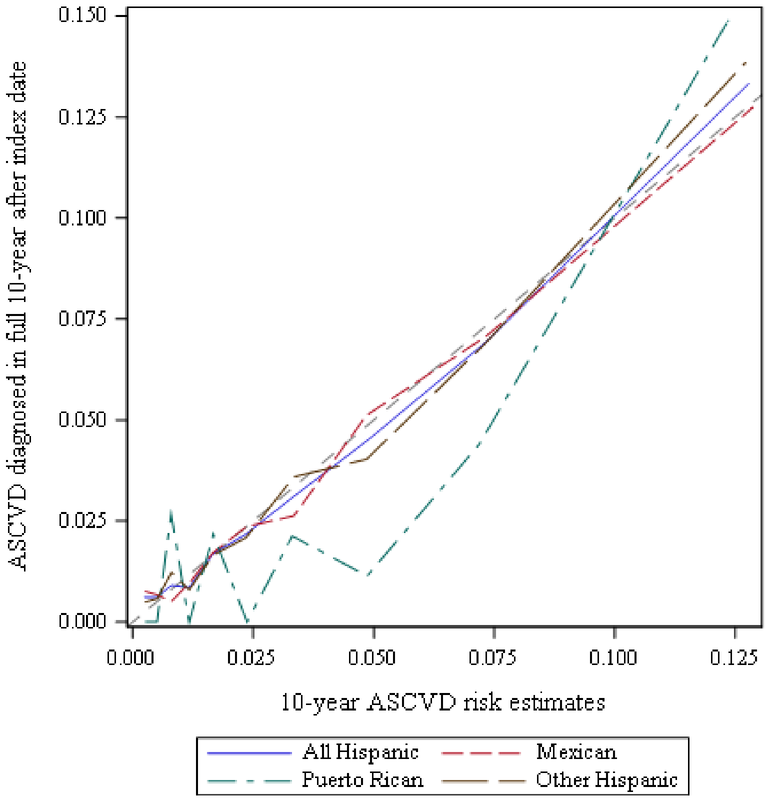

© 2025 Yan X et al. *JAMA Cardiology*.

eFigure 9. Calibration curve for HF, stratified by race/ethnicity group

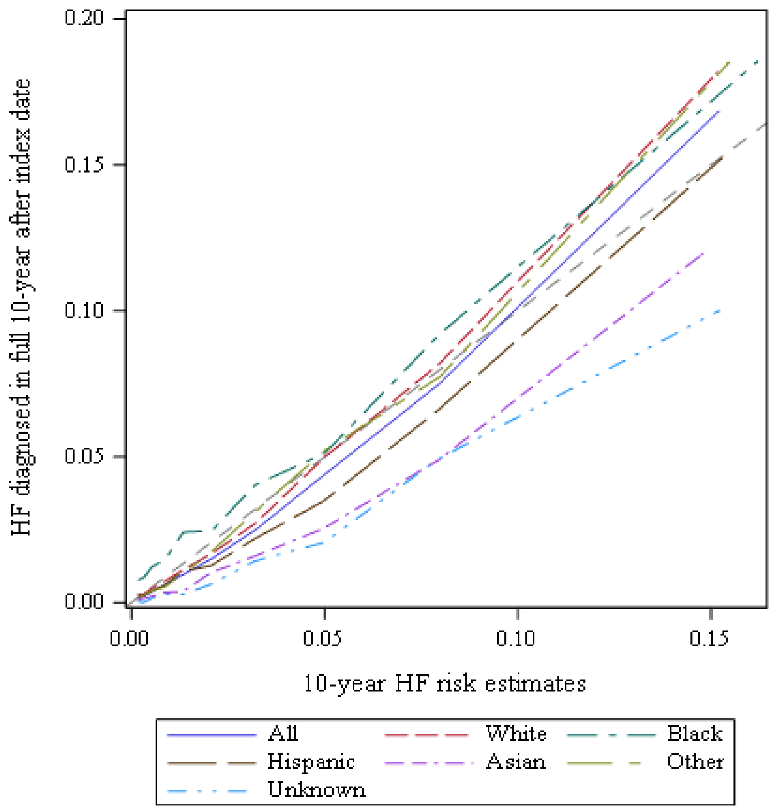

eFigure 10. Calibration curve for HF, stratified by Asian subgroups

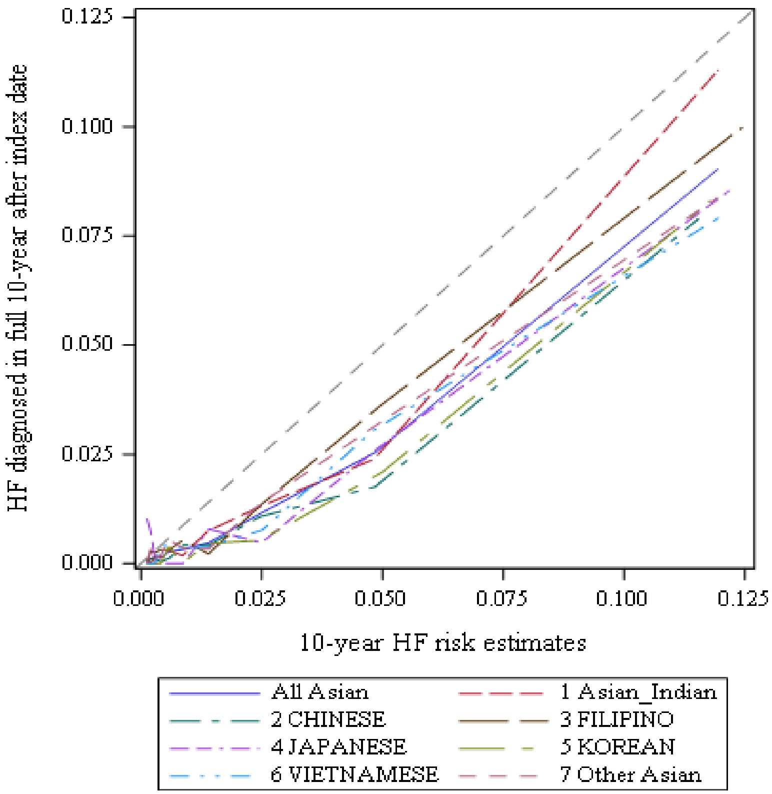

© 2025 Yan X et al. *JAMA Cardiology*.

eFigure 11. Calibration curve for HF, stratified by Hispanic subgroups

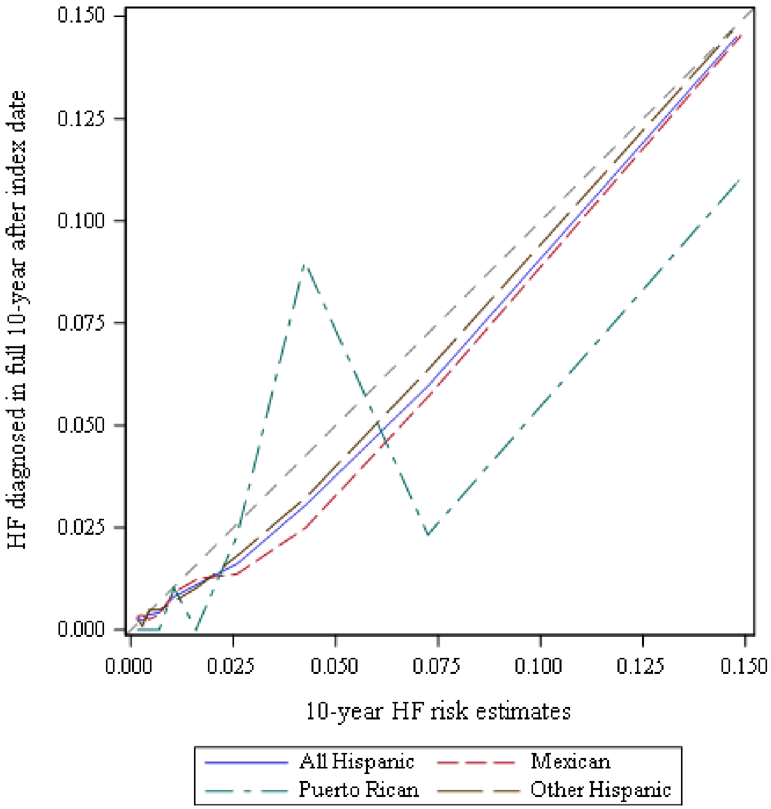

© 2025 Yan X et al. *JAMA Cardiology*.
